# Theoretical-Experimental Study of Magnetic and Optical Properties of Co-Doped BiFeO**_3_**

**DOI:** 10.1021/acsomega.6c01825

**Published:** 2026-08-21

**Authors:** H’Linh Hmŏk, José Mario Galicia Hernández, Rodrigo Ponce Perez, Subhash Sharma, Oscar Raymond Herrera

**Affiliations:** † SECIHTI-Centro de Nanociencias y Nanotecnología, 87793 Universidad Nacional Autónoma de México, Apdo. Postal 14, Ensenada, Baja California 22860, México; ‡ Centro de Nanociencias y Nanotecnología, Universidad Nacional Autónoma de México, Apdo. Postal 14, Ensenada, Baja California 22860, México

## Abstract

This study investigates the effects of cobalt doping on the structural, magnetic, electronic, and optical properties of BiFeO_3_ using density functional theory (DFT) simulations. A 2 × 2 × 2 supercell containing 80 atoms was constructed, and nine distinct Co–Co configurations were systematically evaluated, considering different dopant separations, local spin orientations, and global magnetic orderings (antiferromagnetic, AFM, and ferromagnetic, FM). The BiFe_0.875_Co_0.125_O_3_ solid solution is found to be most stable when the two Co ions are separated by 5.637 Å. The results show that Co doping effectively narrows the band gap of BiFeO_3_, enhancing its optical absorption in the visible and near-infrared regions. New electronic states associated with the Co dopants are introduced near the Fermi level, leading to additional optical transitions at lower energies, 1.25 and 1.75 eV, together with a reduction of the main band gap to 2.55 eV. Furthermore, Co incorporation induces ferrimagnetic behavior with a total magnetic moment of 1.879 μ_B_ per 2 × 2 × 2 supercell (16 formula units). The first-principles calculations show qualitative agreement with previous experimental reports. Overall, these results highlight Co-doped BiFeO_3_ as a viable candidate for optoelectronic and spintronic applications, showcasing enhanced optical properties and structural stability.

## Graphical abstract

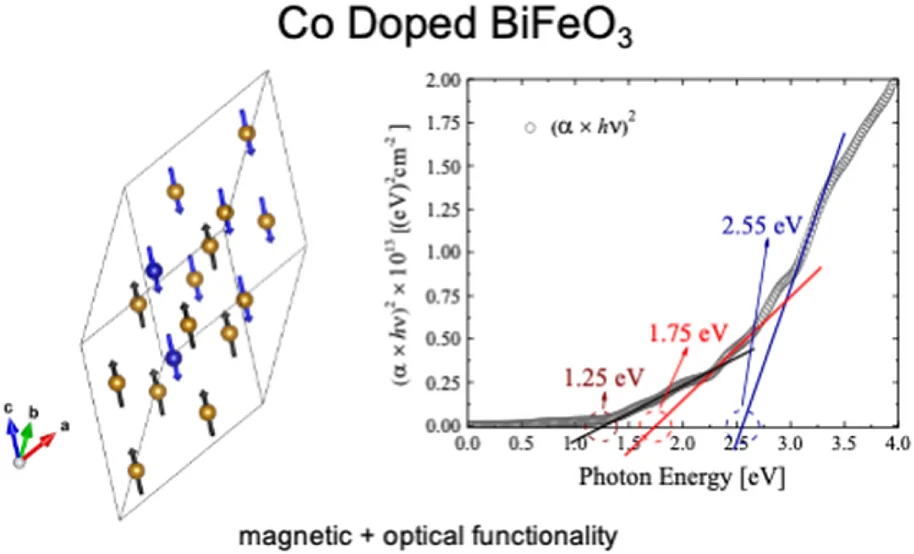

## 1 Introduction

BiFeO_3_ (BFO) compound is known as a promising candidate for applications in data storage, spin valves, spintronics, and sensors.^1,2^ BFO bulk is a multiferroic compound that presents simultaneously ferroelectric and antiferromagnetic properties at room temperature with a Curie temperature of T_C_ = 1103 K and a Néel temperature of T_N_ = 643 K. The crystal structure of BFO at room temperature is rhombohedral, belonging to the *R3c* space group.

Recently, there has been increasing interest in using BFO for photovoltaic applications due to the coexistence of its ferroelectric and narrow band gap properties.^3,4^ There are many reports of direct band gap values between 2.3 and 2.8 eV, while its indirect band gap values are reported to be between 0.4 and 1.0 eV smaller than the direct band gaps.^5^ However, there have been efforts to further reduce this band gap to take advantage of visible light absorption through the use of chemical substitutions. Gao et al. display that the band gap of BFO is decreased to 1.42 eV after doping by substituting the Bi site with Ca 10 at. %.^6^ Grover et al. show that the band gap is reduced from 2.9 to 2.1 eV for BiFe_0.938_Co_0.062_O_3_ using the density functional theory (DFT).^7^ On the other hand, there are some reports about the magnetic properties study of doped BFO. Pugaczowa-Michalska and Kaczkowski studied the solid solutions Bi_0.875_RE_0.125_FeO_3_ (RE = La, Ce, Pr, Nd) based on DFT calculations showing that the magnetization increases with the number of 4*f* electrons when Bi is substituted with rare-earth ions such as Ce, Pr, and Nd.^8^ Using first-principles calculations, Wang et al. show that substituting only one N atom at the O site can introduce a total magnetic moment of 1 μ_*B*_ in BFO,^9^ while Rong et al.^10^ report that Co-doped BFO produces a magnetic moment of −1.0 μ_*B*_ and displays a half-metallic behavior. Additionally, Sui et al. show that the net magnetic moment by unit cell was evaluated to be 0.93 μ_*B*_ for BiFe_0.83_Co_0.17_O_3_, which may be attributed to Fe^3+^–O^2–^–Co^3+^ superexchange interactions.^11^

On the other hand, the previous experimental report^12^ shows that BiFe_0.9_Co_0.1_O_3_ solid solution exhibits ferrimagnetic behavior, and the UV–Vis–NIR measurements reveal three band gap values (1.21, 1.85, and 2.98 eV), which are associated with Co ions and oxygen vacancies. Based on these experimental results obtained for BiFe_0.9_Co_0.1_O_3_ solid solution, we were motivated to explore and understand its optical and magnetic properties from first-principles calculations. Although previous DFT studies on Co-doped BiFeO_3_ have reported band gap reduction and magnetic modifications,^7,11^ a systematic investigation of dopant–dopant configurational effects—particularly their spatial separation and magnetic coupling—remains lacking. In this work, we address this gap by examining multiple Co–Co configurations with varying separation and magnetic alignment, thereby enabling the construction of a quantitative configurational stability map. Furthermore, we perform a detailed optical analysis and directly compare the calculated spectra with experimental data, which allows a physical interpretation of the observed multiband gap features. This combined configurational and theory–experiment approach provides a more comprehensive understanding of the electronic, magnetic, and optical behavior of Co-doped BiFeO_3_.

## 2 Computational and Experimental Details

First-principles density functional theory (DFT) calculations were performed using the Vienna Ab initio Simulation Package (VASP).^13,14^ Electron–ion interactions were described using the projector augmented-wave (PAW) method, with a plane-wave kinetic energy cutoff of 470 eV. Exchange-correlation effects were treated using the RSCAN meta-generalized gradient approximation (meta-GGA) functional.^15^ To improve the description of the localized Fe-3*d* and Co-3*d* electrons, the simplified rotationally invariant DFT+U approach proposed by Dudarev et al.^16^was employed, using an effective Hubbard parameter of U = 5 eV for Fe and Co. Although meta-GGA functionals such as RSCAN generally provide improved structural and energetic properties compared with conventional GGA functionals, residual self-interaction errors remain for localized transition-metal 3*d* states. The Hubbard correction, therefore, enhances the description of electronic localization, magnetic ordering, and band gap values in Co-doped BiFeO_3_. To ensure convergence of total energies, a 4 × 4 × 4 k-point mesh was employed for structural relaxation and an 8 × 8 × 8 mesh for electronic structure calculations. Tests with denser k-point grids yielded energy variations below a few meV per supercell, confirming that the calculated energy differences are well converged. The total energy convergence criterion was fixed at 10^–6^ eV and the Hellmann–Feynman forces were relaxed below 0.01 eV/Å.

For describing the optical properties, we computed the frequency-dependent dielectric function by applying the random phase approximation (RPA) and using the independent particle approximation (IP) for computation of the dielectric tensor. From these calculations, the real (ε_1_) and imaginary (ε_2_) parts of the dielectric function were obtained. We used a cutoff energy of 500 eV for the wave function expansion and a corresponding *k*-points mesh of 12 × 12 × 12 for the sampling of the first Brillouin zone. Within the Dudarev DFT+U framework,^15,16^ the Hubbard U parameter is treated as an effective, property-dependent quantity. A value of U = 5 eV was employed for Fe/Co 3*d* states to accurately describe ground-state properties, including total energies and magnetic ordering. For optical properties, calculated using the dielectric function within the random phase approximation (RPA) and independent-particle framework, the same U value leads to a systematic overestimation of band gaps due to the absence of excitonic and many-body effects. Therefore, reduced values of U_Fe_ = 2.5 eV and U_Co_ = 1.5 eV were adopted to obtain more realistic band edge positions and optical transition energies. This approach is widely employed in DFT+U studies of correlated oxides.^17,18^

Once having the real and imaginary parts of the dielectric function, we calculated the absorption coefficient α spectrum as a function of the frequency ω using the equation:

1 $$\alpha \left(\omega \right)=\frac{\sqrt{2}\omega }{c}{\left\{{[{\varepsilon_{1}}^{2}\left(\omega \right)+{\varepsilon_{2}}^{2}\left(\omega \right)]}^{1/2}-\varepsilon_{1}\left(\omega \right)\right\}}^{1/2}$$

where *c* is the speed of light in the vacuum.

Finally, we used the Tauc method^19^ to estimate the electronic band gap from the calculated optical absorption spectra. This method assumes that the absorption coefficient α is related to the electronic band gap *E*_*g*_ by the following equation:

2 $${\left(\alpha \times h\nu \right)}^{\gamma }=B\left(h\nu -E_{g}\right)$$

where *h* is the Planck’s constant, ν is the photon frequency, and *B* is a proportionality constant. Thus, the spectrum of the dielectric functions and α as a function of ω can be illustrated as a function of the photon energy using the relation ν = ω/2π.

The work focuses on zero-temperature DFT predictions. At finite-temperature effects or under external strain, we expect slight modifications in lattice parameters, magnetic moments, band gaps, and optical transitions. In addition, spin–orbit coupling (SOC) was not included in the present calculations. Although Bi is a heavy element, previous first-principles studies have shown that in BiFeO_3_, the valence band maximum is primarily composed of O-2*p* states, while the conduction band edge is dominated by Fe-3*d* states with Bi-6*p* states located at higher energies.^20,21^ Therefore, the electronic states relevant to the band gap and magnetic properties are mainly of 3*d* character, for which SOC effects are comparatively small. SOC is known to primarily influence magnetic anisotropy and induce small band splittings (on the order of meV) without significantly affecting band gap values or magnetic ordering trends in such systems. Thus, the main conclusions of this work are not expected to be qualitatively affected by the inclusion of SOC.

The synthesis procedure of Co-doped BFO with the stoichiometry BiFe_0.90_Co_0.10_O_3_, X-ray diffraction (XRD) analysis, and UV–vis-NIR measurements have been previously reported in detail in ref 12 and are therefore not repeated here. Only the key results relevant to the present theoretical analysis are summarized here. The XRD data confirm the rhombohedral structure with *R3c* symmetry in the hexagonal cells (Figure 1a). The Rietveld refinement confirms that the rhombohedral *R3c* phase is the predominant crystalline phase in the synthesized sample, while a minor secondary phase described by the *I23* structural model is also present, as reported in ref 12. Consequently, the magnetic and optical properties discussed in this work are expected to be governed primarily by the Co-doped BiFeO_3_ phase. Although a small contribution from the impurity phase cannot be entirely excluded, its low abundance is unlikely to affect the principal experimental trends or the interpretation of the first-principles calculations.

**Figure 1:**
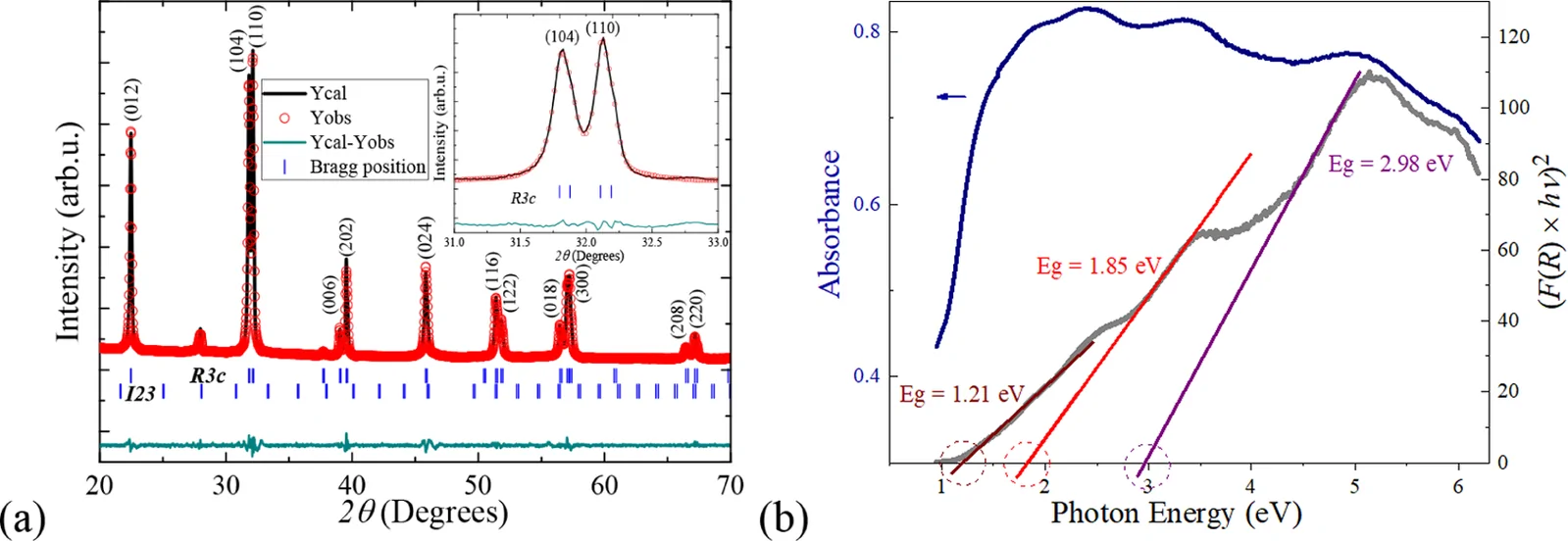
(a) Powder X-ray diffraction pattern (red circles) of the Co-doped BiFeO_3_ sample and the Rietveld refinement result (black solid line) using the rhombohedral *R3c* symmetry with a hexagonal cell for BiFe_0.90_Co_0.10_O_3_ and the *I23* model for the impurity phase. The inset shows the fit details for the peaks of the (104) and (110) planes. (b) UV–vis-NIR absorbance spectrum (blue line) obtained from diffuse reflectance and the Kubelka–Munk plot (gray line) of the quantity (*F*(*R*) × *h*ν)^2^ as a function of photon energy. Adapted with permission from Sharma, S.; et al. *J. Mater. Chem. C* **2025**, 13, 9138–9147. Copyright 2025 Royal Society of chemistry.

Figure 1b shows the UV–vis-NIR absorbance curve obtained by diffuse reflectance for the Co-doped BiFeO_3_ sample, reported in the previous work.^12^ The spectrum illustrates a strong absorption coming from the near-infrared region (1.03 eV) and three well-defined peaks around 2.38, 3.35, and 4.96 eV, suggesting an overlap of different absorption processes associated with a complex band structure with different band gap energy (*E*_*g*_) values. The Kubelka–Munk function *F*(*R*) and the Tauc plot of the quantity (*F*(*R*) × *h*ν)^γ^ as a function of the photon energy (*h*ν) were used to obtain the *E*_*g*_ values for direct (if γ = 2) or indirect (if γ = 0.5) allowed transitions.^22,23^ Surprisingly, as illustrated in Figure 1b, three linear regions in the (*F*(*R*) × *h*ν)^2^ vs *h*ν curve with different absorption edges are observed. Thus, the direct *E_g_* values of 1.21, 1.85, and 2.98 eV were calculated from the intersection point of the *h*ν axis with the tangent line to the inflection point of each linear region.^12^

## 3 Results and Discussion

### 3.1 Structural and Magnetic Properties

Pristine BFO has a rhombohedral structure belonging to space group *R3c* with an atomic content by cell of 2 Bi, 2 Fe, and 6 O ions. A 2 × 2 × 2 supercell (80 atoms, comprising 16 Bi, 16 Fe, and 48 O ions; see Figure 2a) was employed as a balance between computational cost and physical representativeness. By substituting two Fe ions with two Co ions, a Co concentration of 12.5% is achieved, corresponding to the composition of BiFe_0.875_Co_0.125_O_3_. This value is close to the experimental concentration (10%) and enables the investigation of multiple Co–Co separations within a consistent periodic framework. The aim of this study is to analyze dopant–dopant interaction trends rather than the dilute limit, for which this supercell is particularly appropriate.

**Figure 2:**
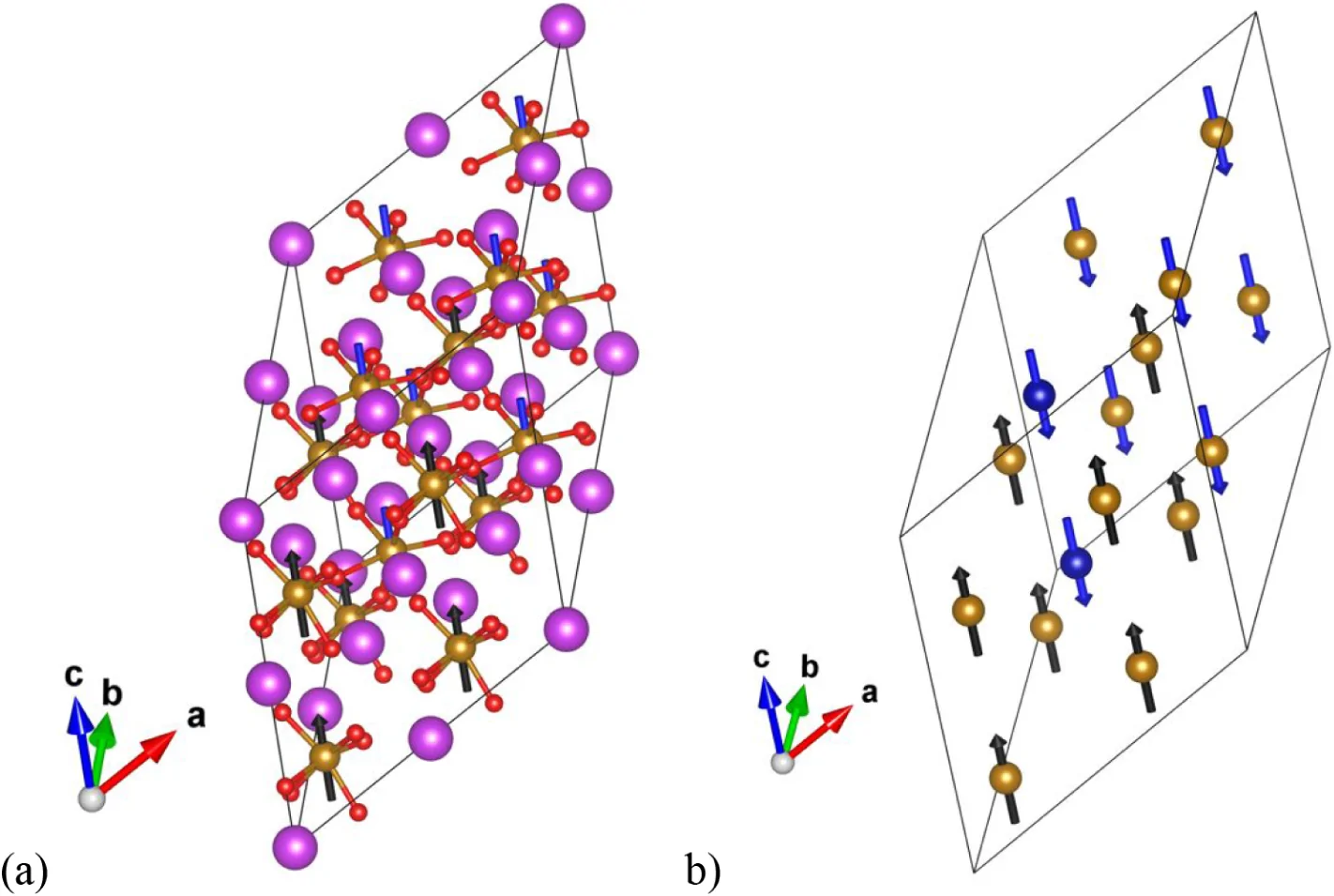
Supercell of the (a) pristine BiFeO_3_ and (b) the most stable configuration of BiFe_0.875_Co_0.125_O_3_ solid solution (O and Bi ions were removed to better illustrate the magnetic moments of Co and Fe ions). Color scheme: Bi (purple), O (red), Fe (brown), Co (blue). The black-colored vectors are antiparallel to the blue-colored vectors.

Table 1 reports the values of the relative system’s energy and the total magnetization by unit supercell, calculated for nine different possible configurations considered when substituting two Fe ions by two Co ions (structural models and magnetic moments for all nine configurations are provided in the Supporting Information), taking into account different distances between the two Co ions and their magnetic moment orientations (parallel or antiparallel). Antiferromagnetic and ferromagnetic ordering of all magnetic moments are considered as well. For example, in Configuration 4, the two Co spins are aligned parallel to each other but antiparallel to the surrounding Fe spins, resulting in an overall antiferromagnetic configuration. The system’s energy was used as a criterion of stability of the BiFe_0.875_Co_0.125_O_3_ solid solution, i.e., the configuration with less energy value is the most stable. The relative system’s energies for all nine configurations are summarized in column 5 of Table 1. Among the considered arrangements, configuration 4 (Co–Co distance = 5.637 Å with antiferromagnetic ordering) is identified as the most stable and is therefore taken as the reference with zero energy. All other configurations are reported relative to this value. Although some AFM configurations exhibit small energy differences, the ground-state configuration is consistently identified, and the energy variation as a function of Co–Co distance displays systematic trends. This confirms that the results are physically meaningful rather than numerical noise. From this comparison, the remaining AFM configurations are higher in energy by approximately 0.008–0.776 eV, while all FM configurations are significantly less favorable with energies more than 3 eV above the ground state. These results indicate that the BiFe_0.875_Co_0.125_O_3_ solid solution is most stable when both Co ions are separated by a distance of 5.637 Å in the cell; it means that the distance between Co ions is neither the furthest nor the nearest but is intermediate and fulfilling with a global antiferromagnetic ordering. Furthermore, the magnetic moments of the two Co ions tend to maintain a parallel alignment within the AFM host magnetic structure.

**Table 1:** Values of the Relative System Energy and the Total Magnetization, Calculated for Different Possible Configurations (Conf.) of the Distance between Co Ions, Their Magnetic Moment Orientations (MMO), and the System Magnetic Ordering (SMO)^a^

Conf.	Co–Co distance (Å)	MMO of Co ions	SMO	System energy (eV)	Total magnetization (μ_B_)
1	3.968	AP	AFM	0.008	–0.006
2	3.968	P	AFM	0.350	7.842
3	3.968	P	FM	3.002	74.599
4	5.637	P	AFM	0.000	1.879
5	5.637	AP	AFM	0.357	9.766
6	5.637	P	FM	3.010	76.384
7	6.934	AP	AFM	0.034	0.012
8	6.934	P	AFM	0.776	7.648
9	6.934	P	FM	3.024	76.363

a Here, P (parallel) and AP (antiparallel) refer to the relative spin alignment between the two Co atoms only, while the global magnetic ordering (AFM or FM) refers to the overall spin arrangement of the system, primarily defined by the Fe sublattice.

The magnetic behavior of Co-substituted BiFeO_3_ originates from the disruption of the G-type antiferromagnetic order of pristine BFO due to the introduction of inequivalent magnetic sublattices. While Fe^3+^ ions (3d^5^, high spin) interact through antiferromagnetic Fe–O–Fe superexchange, Co substitution introduces additional Fe–O–Co exchange pathways. According to the Goodenough–Kanamori rules, the Fe^3+^–O–Co^3+^ superexchange interaction remains predominantly antiferromagnetic. However, the different magnetic moments of Fe and Co ions prevent full compensation, giving rise to a ferrimagnetic ground state with a finite net magnetization. Figure 2a and 2b show the supercell of pristine BFO and the most stable configuration of BiFe_0.875_Co_0.125_O_3_ solid solution with the two Co ions separated by a distance of 5.637 Å, respectively. Henceforth, we only use these configurations to study magnetic, electronic, and optical properties.

For the most stable supercell configuration of BiFe_0.875_Co_0.125_O_3_, the Co ions’ magnetic moments (3.248 μ_B_) are lower than those of the Fe ions (4.373 μ_B_). Such a difference between magnetic moments establishes a total magnetization per 2 × 2 × 2 supercell (16 formula units of BiFeO_3_) of 1.879 μ_B_, i.e., 2.1 A·m^2^·kg^–1^. To express 1.879 μ_B_ in units of A·m^2^·kg^–1^, the magnetic moment was first converted from μ_B_ to A·m^2^ and then normalized by the total mass of the supercell, calculated using the molar mass of Bi, Fe, O, and Co atoms in the supercell. The calculated magnetization of approximately 2.1 A·m^2^·kg^–1^ is about six times larger than the experimental value of approximately 0.35 A·m^2^·kg^–1^. This discrepancy is expected because the present DFT calculations describe an idealized, perfectly ordered collinear magnetic configuration at 0 K within a finite 2 × 2 × 2 supercell. In contrast, the synthesized Co-doped BiFeO_3_ is characterized by several mechanisms that reduce the net magnetization, including spin canting associated with the Dzyaloshinskii–Moriya interaction, defect-related magnetic disorder and local compositional fluctuations, finite crystallite size and domain effects, and other microstructural inhomogeneities. Consequently, the DFT results should be regarded as an upper bound for the magnetization of an idealized crystalline model. Despite the quantitative difference in magnitude, both theory and experiment consistently demonstrate that Co substitution transforms the antiferromagnetic ordering of pristine BiFeO_3_ into a ferrimagnetic ground state. Accordingly, the comparison between theory and experiment should be interpreted as qualitative rather than quantitative.

Table 2 shows the lattice parameters, angles, and unit cell volume of the supercell obtained for pristine BFO and the most stable configuration of the BiFe_0.875_Co_0.125_O_3_ solid solution. The lattice parameters and angles are very similar to those experimental values measured for BiFe_0.9_Co_0.1_O_3_.^12^ As can be seen, the substitution of Fe by Co in the pristine BFO is almost without structural change, which is attributed to the fact that the ionic radii of Co^3+^ and Fe^3+^ ions in 6-fold coordination are 0.55 Å.^24^

**Table 2:** Values of Supercell Rhombohedral Lattice Parameters and Unit Cell Volume of Pristine BiFeO_3_ and BiFe_0.875_Co_0.125_O_3_ Solid Solution, in Comparison with Those Experimental Values Reported for BiFe_0.9_Co_0.1_O_3_.^12^

	BiFeO_3_	BiFe_0.875_Co_0.125_O_3_	BiFe_0.9_Co_0.1_O_3_ ^12^
*a* = *b* = *c* (Å)	11.3258	11.3146	11.248(6)
α = β = γ(°)	59.42	59.42	59.3(7)
V (Å^3^)	1013.82	1010.81	992.0(0)

### 3.2 Electronic Properties

Figure 3a shows the partial density of states (PDOS), and Figure 3b illustrates the band structure of pristine BFO. As can be seen in both figures, the electronic states of *spin-up* and *spin-down* channels are identical, indicating, as expected, that the pristine BFO behaves as an antiferromagnetic material. Additionally, it exhibits a band gap value of 2.92 eV, which is in good agreement with the experimental values reported in ref 2. Moreover, the band gap of this compound is established mainly by the contribution of the O 2*p* orbitals at the maximum of the valence band and the Fe 3*d* orbitals in the minimum of the conduction band.

**Figure 3:**
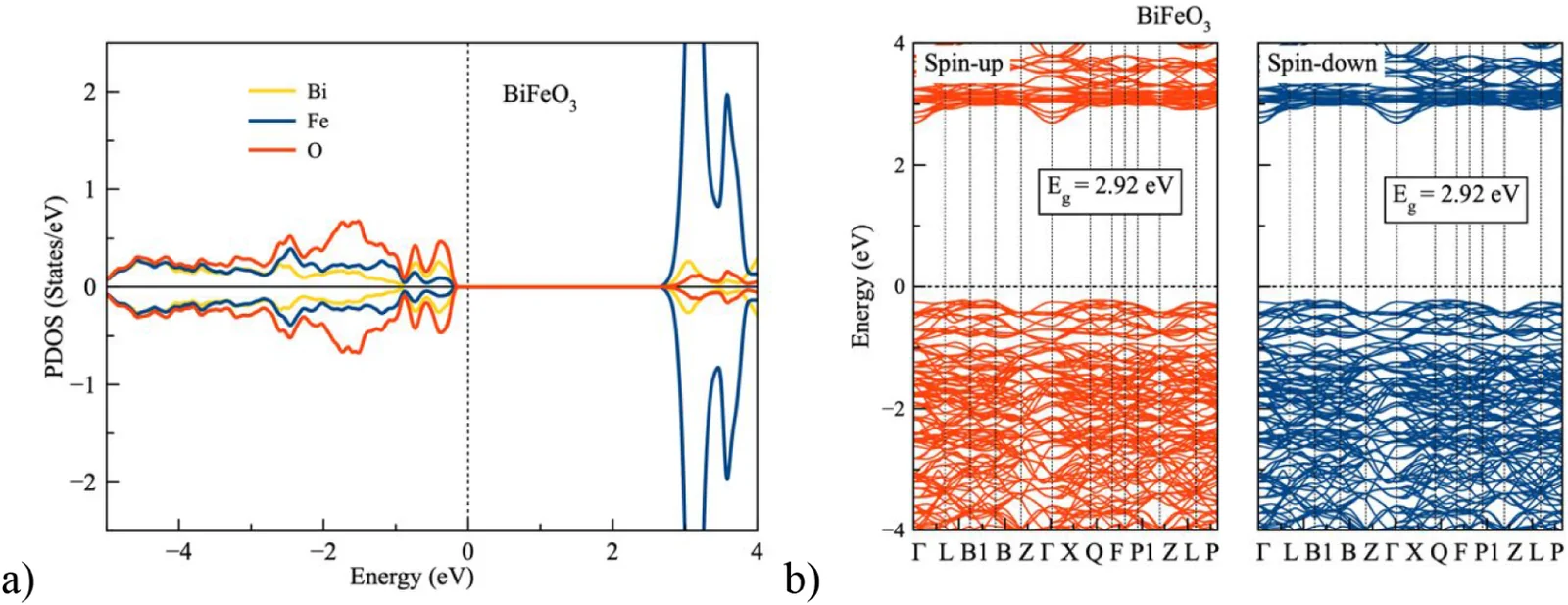
(a) Spin-polarized projected density of states (PDOS) of pristine BiFeO_3_. Positive and negative values correspond to the spin-up and spin-down channels, respectively. The projected contributions from the Bi, Fe, and O atomic orbitals reveal the electronic states that form the valence and conduction bands. (b) Spin-polarized band structure of pristine BiFeO_3_ for the spin-up and spin-down channels. The energy is referenced to the Fermi level, which is set to 0 eV.

When the pristine BFO is doped with Co 12.5 at. % in substitution of Fe ions, the electronic properties of the compound are significantly affected. As shown in Figure 4a, the majority contribution to the density of states near the minimum of the conduction band corresponds with the appearance of Co 3*d* orbitals on the *spin-up* channel. This Co-3*d* state contribution coupled with a minority of O-2*p* states establishes an asymmetry in the PDOS profiles, especially on the conduction band. As a consequence of this, Figure 4b exhibits a reduction of the *spin-up* band gap from 2.92 to 2.13 eV, while the *spin-down* band gap remains practically unchanged compared to the pristine BFO electronic structure (Figure 3). The computed electronic band gap values of the Co-doped system are in good agreement with the experimental values reported in ref 12. Furthermore, previous DFT study reports similar trends where the band gap is reduced from 2.9 to 2.1 eV within the same solid solution.^7^ In addition, the coexistence of spin-polarized electronic states and ferrimagnetic order enabled spintronic devices such as spin filters or spin-photovoltaic elements, where spin-dependent carrier transport is required.

**Figure 4:**
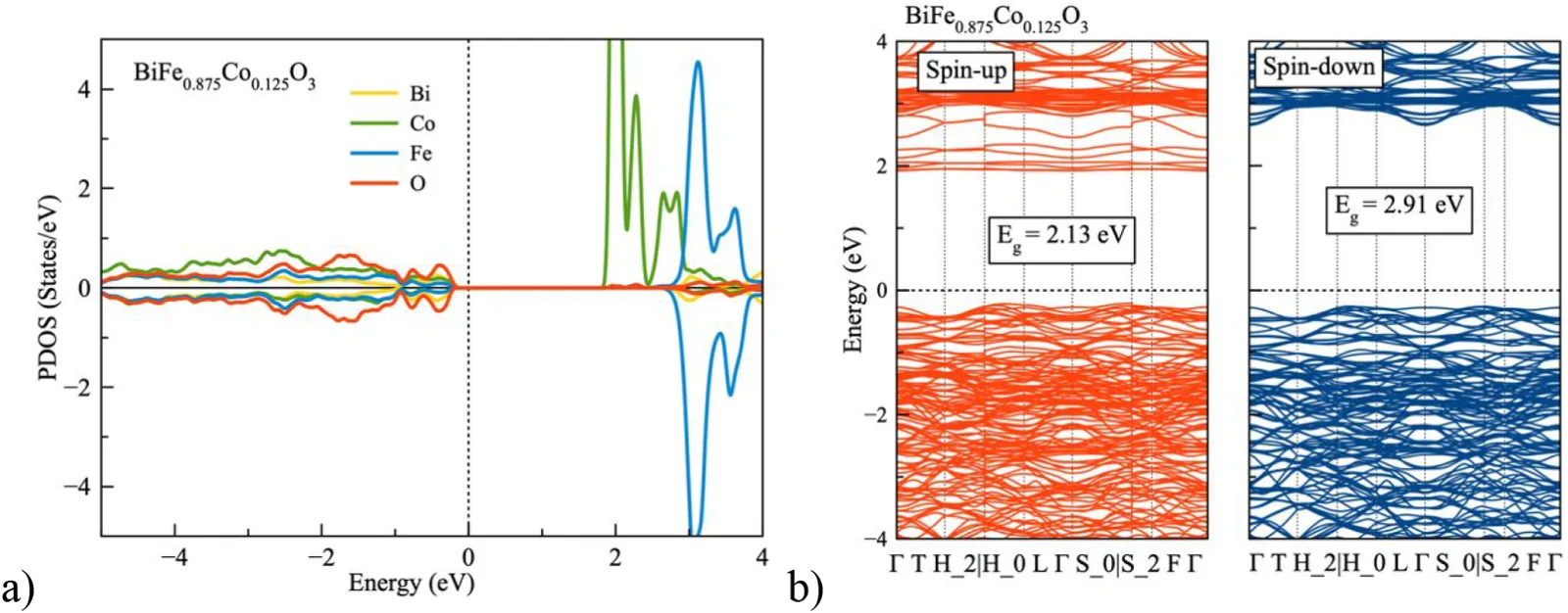
(a) Spin-polarized projected density of states (PDOS) for Co-doped BiFeO_3_. Positive and negative values correspond to the spin-up and spin-down channels, respectively. The projected contributions from Bi, Fe, Co, and O atomic orbitals illustrate the electronic states responsible for the valence band, conduction band, and the Co-induced localized states near the band edges. (b) Spin-polarized band structure of Co-doped BiFeO_3_ for the spin-up and spin-down channels. The energy is referenced to the Fermi level, which is set to 0 eV.

### 3.3 Optical Properties

In order to have a clearer explanation of the UV–vis-NIR absorbance spectrum and the three band gap values presented in Section 2, we used the DFT+U/RPA-IP approach as described in Section 2. For describing the electronic transitions from valence to conduction band, it is useful to analyze the imaginary part of the dielectric function, as the peaks of this response function are associated with the direct electronic transitions. In this way, the theoretical results of the computed imaginary part of the dielectric function obtained for the pristine BiFeO_3_ and doped BiFe_0.875_Co_0.125_O_3_ systems are illustrated in Figure 5a and Figure 6a, respectively.

**Figure 5:**
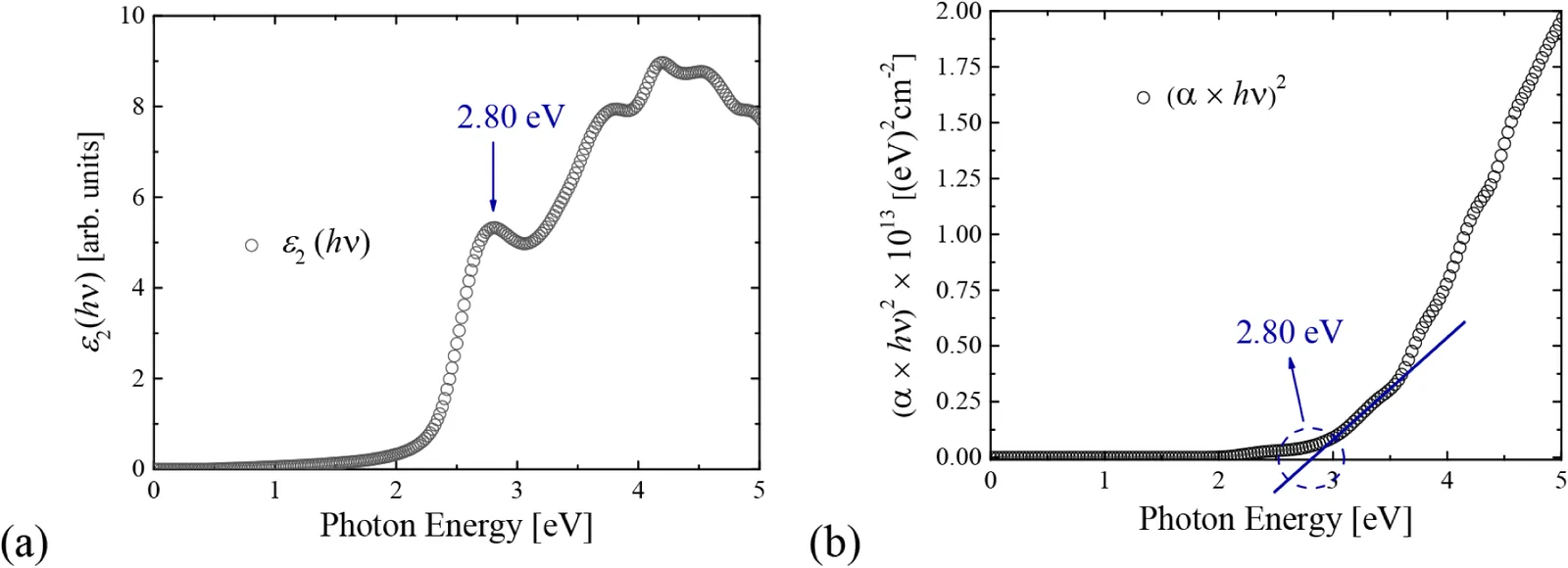
Optical response calculated for the pristine BiFeO_3_: (a) the imaginary part of the dielectric function and (b) the Tauc plot of the (α × *h*ν)^2^ as a function of the photon energy *h*ν. α is the absorption coefficient.

**Figure 6:**
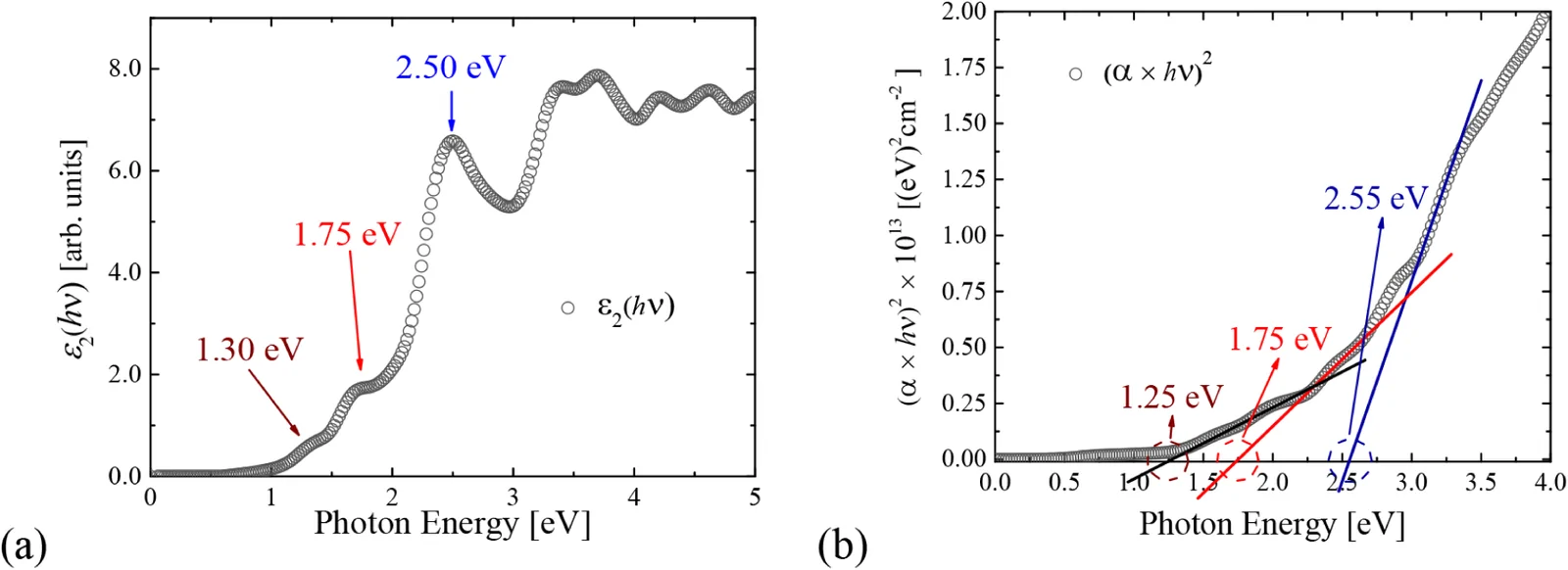
Optical response calculated for the doped BiFe_0.875_Co_0.125_O_3_ solid solution: (a) the imaginary part of the dielectric function and (b) the Tauc plot of the (α × *h*ν)^2^ as a function of the photon energy *h*ν. α is the absorption coefficient.

The optical response of the system was observed to be isotropic for the *x*- and *y*-directions (as we obtained the same results for the dielectric function imaginary part), and a very weak response was observed along the *z*-direction. Thus, the contribution for the *z*-direction was considered negligible, and it was not taken into account. So, the plots indicate the optical response along the *x*- and *y*-directions.

It should be emphasized that the present DFT model explicitly considers only Co substitution at the Fe site and does not account for the oxygen vacancies. The objective of these calculations is to isolate the intrinsic effect of Co incorporation on the electronic structure and the optical response of BiFeO_3_. By contrast, the experimental samples may contain a finite concentration of oxygen vacancies, which have previously been suggested to contribute to the observed optical absorption features (see ref 12).

In Figure 5a, we can identify a peak located at 2.8 eV for the pristine BFO, which is related to direct transitions at the Γ point, ascribed to the direct electronic band gap. Once computing the absorption coefficient spectrum using eq 1, we estimated the value of the band gap by using the Tauc method (eq 2 for direct transitions), and as shown in Figure 5b, the band gap is 2.8 eV, as confirmed by the obtained value from the dielectric function. Our predicted theoretical value is in correspondence with the calculated band gap value of 2.92 eV reported in the previous section and in good agreement with previous works, in which they establish that the value at room temperature ranges from 2.3 to 2.8 eV.^5^ The small difference between the electronic and optical band gap is related to the fact that the optical part is associated with the absorption onset, i.e., the absorption edge, which is generally at lower energies than the electronic band gap.

Figure 6 depicts the results for the doped BiFe_0.875_Co_0.125_O_3_ system. From the imaginary part of the dielectric function (Figure 6a), it is noticeable that there are two small peaks located at low energies, centered around 1.30 and 1.75 eV. On the basis of the results regarding the electronic properties, we can establish that both peaks are attributed to the Co-dopant atoms. As observed in Figure 4a, the calculation of the projected density of states confirms that the first electronic states above the Fermi level arise from the contribution of Co orbitals. In other words, the edge of the conduction band is formed by Co orbitals, and the electronic transitions from the valence to the conduction band will predominantly occur from O orbitals to Co orbitals. Thus, we can conclude that the dopant atoms contribute electronic states that narrow the band gap of pristine BFO, permitting the electronic transitions predicted by the two low-energy peaks in the imaginary part of the dielectric function.

On the other hand, a third peak centered at 2.50 eV is observed in the imaginary part of the dielectric function (Figure 6a). This peak is related to the direct electronic transitions to states of the host system (BiFeO_3_), as can be confirmed with the calculation of the projected density of states, in which we can observe that, for higher energies, the main contribution to orbitals comes from the Bi, Fe, and O atoms. We can also conclude that the presence of the dopant shifts the energy value at which this third peak appears, as this peak is located at 2.8 eV in the pristine system (Figure 5a).

Finally, from the dielectric function obtained for the doped BiFe_0.875_Co_0.125_O_3_ system, we calculated the absorption coefficient using eq 1 and applying the Tauc method (eq 2), we obtained the plot of (α × *h*ν)^2^ versus *h*ν, as illustrated in Figure 6b. Thus, the calculated optical spectrum exhibits three characteristic absorption features. The transitions at approximately 1.25 and 1.75 eV correspond to sub-band gap optical transitions involving Co-induced localized electronic states near the conduction-band edge, thereby introducing additional absorption channels at photon energies below the fundamental absorption edge. In contrast, the transition at approximately 2.55 eV is identified as the fundamental optical band gap of Co-doped BiFeO_3_, arising from transitions between the valence-band maximum and the conduction-band minimum. The calculated spectrum thus reproduces the experimentally observed low-energy optical features while providing a microscopic explanation of their electronic origin. Since oxygen vacancies were not explicitly included in the present DFT model, the comparison with the experimental optical spectrum should be regarded as qualitative. It should be noted that the projected density of states (PDOS) and the optical band gaps extracted from Tauc plots probe different physical quantities. The PDOS represents the distribution of electronic states, whereas the optical absorption coefficient depends on the joint density of states and transition matrix elements.^25,26^ The three optical transitions identified at ∼1.25 eV, ∼1.75 eV, and ∼2.55 eV arise from distinct electronic excitation channels rather than from three separate conduction band edges. The low-energy transitions are attributed to O-2*p* → Co-3*d* impurity states introduced near the conduction band edge, while the higher-energy transition corresponds to the main O-2*p* → Fe-3*d*/Bi states of the host lattice. The Co-related states appear as localized features in the PDOS and do not form sharply separated band edges.

It is worth noting that the calculated optical transitions show excellent agreement with experiment at low energies (1.25 eV vs 1.21 eV and 1.75 eV vs 1.85 eV), while the higher-energy transition (2.55 eV vs 2.98 eV) exhibits a larger deviation. This discrepancy in the higher-energy transitions can be attributed to the limitations of the DFT+U approach within the random phase approximation (independent-particle framework), which does not account for electron–hole (excitonic) interactions or many-body quasiparticle corrections.^25,27^ These effects become increasingly important at higher photon energies and can lead to systematic shifts of optical peaks. In contrast, low-energy transitions near the band edge, dominated by localized states (e.g., Co 3*d* and O 2*p*), are generally well described by DFT+U.^28^ Despite the deviation at higher energy, the calculations correctly reproduce the presence of multiple optical transitions and the overall band gap reduction trend. The reduced band gap and enhanced optical absorption upon Co substitution suggest potential for photovoltaic devices and visible-light photodetectors where light-induced charge carriers are critical.

Results indicate that the band gaps of lower energy are very close to the ones reported by the experiment. We observe a larger difference for the last band gap, and this is related to the fact that the optical properties computed within DFT methods are considered more accurate for low energies, i.e., for transitions that occur near the band edges, as band edge dispersions are well described by DFT methods. This is why optical onsets and trends of the dielectric function computed by DFT are in good agreement with experimental results. In summary, the DFT approach is a good theoretical technique to describe low-energy excitations because these transitions involve similar orbitals and the matrix elements vary slowly.

## 4 Conclusions

Based on first-principles calculations, this work presents a systematic investigation of BiFeO_3_ and BiFe_0.875_Co_0.125_O_3_ solid solutions, with particular emphasis on the role of dopant configuration in determining structural, electronic, magnetic, and optical properties. Nine configurations with different Co–Co distances and magnetic ordering were considered. The structure of the BiFe_0.875_Co_0.125_O_3_ solid solution is most stable when Co ions are separated by an intermediate distance of 5.637 Å, resulting in a ferrimagnetic ground state. Although the calculated magnetization exceeds the experimental value due to the idealized collinear DFT model, both theoretical and experimental results consistently predict the emergence of ferrimagnetism upon Co substitution. Accordingly, the comparison between the calculated and measured magnetic moments should be interpreted as qualitative rather than quantitative. Additionally, new electronic states associated with the Co dopant in the BiFeO_3_ were introduced near the Fermi level, leading to additional optical transitions at lower energies, 1.25 and 1.75 eV, and a noticeable reduction of the main band gap to 2.55 eV is also observed. The calculated optical response enables a direct interpretation of the experimentally observed multiple absorption features, linking them to Co-induced electronic states and intrinsic host transitions. These findings demonstrate that the integration of configurational analysis with theory-experiment correlation provides a deeper understanding of the multifunctional behavior of Co-doped BiFeO_3_. This analysis suggests that the BiFe_0.875_Co_0.125_O_3_ solid solution could serve as a promising candidate for potential optoelectronic and spintronic applications.

## Supplementary Material



## Notes

Supercell of nine configurations of the BiFe_0.875_Co_0.125_O_3_ solid solution (Figure S1); magnetic moments of all ions of pristine BiFeO_3_ and nine distinct configurations the BiFe_0.875_Co_0.125_O_3_ solid solution (Table S1) (PDF)
