# Supplementary material for: Theoretical-Experimental Study of Magnetic and Optical Properties of Co-Doped BiFeO 3
Source: ACS Omega. 2026 Aug 21;11(34):50831–8. doi: 10.1021/acsomega.6c01825 (PMC13625079; doi:10.1021/acsomega.6c01825)
Supplement: Supplementary file 1 [file ao6c01825_si_001.pdf]

# Theoretical-experimental study of magnetic and optical properties of Co doped $BiFeO_3$


H'Linh Hmŏk[1,*], José Mario Galicia Hernández[2], Rodrigo Ponce Perez[2], Subhash Sharma[1], Oscar Raymond Herrera[2,*]

*[1]SECIHTI-Centro de Nanociencias y Nanotecnología, Universidad Nacional Autónoma de México, Apdo. Postal 14, Ensenada 22860, Baja California, México*

*[2]Centro de Nanociencias y Nanotecnología, Universidad Nacional Autónoma de México, Apdo. Postal 14, Ensenada 22860, Baja California, México*

The corresponding author's e-mails: hlinh.hmok@gmail.com and raymond@ens.cnyn.unam.mx


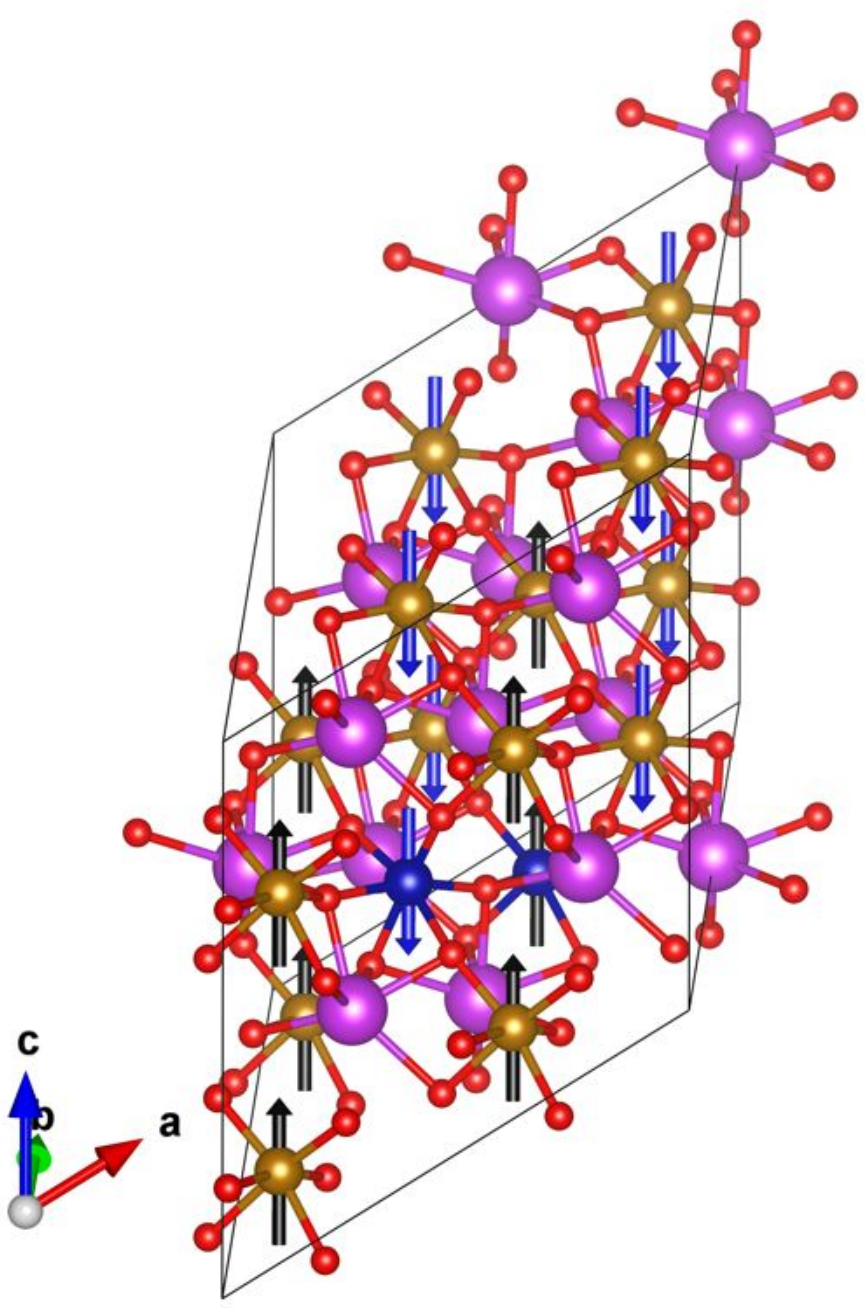


a)

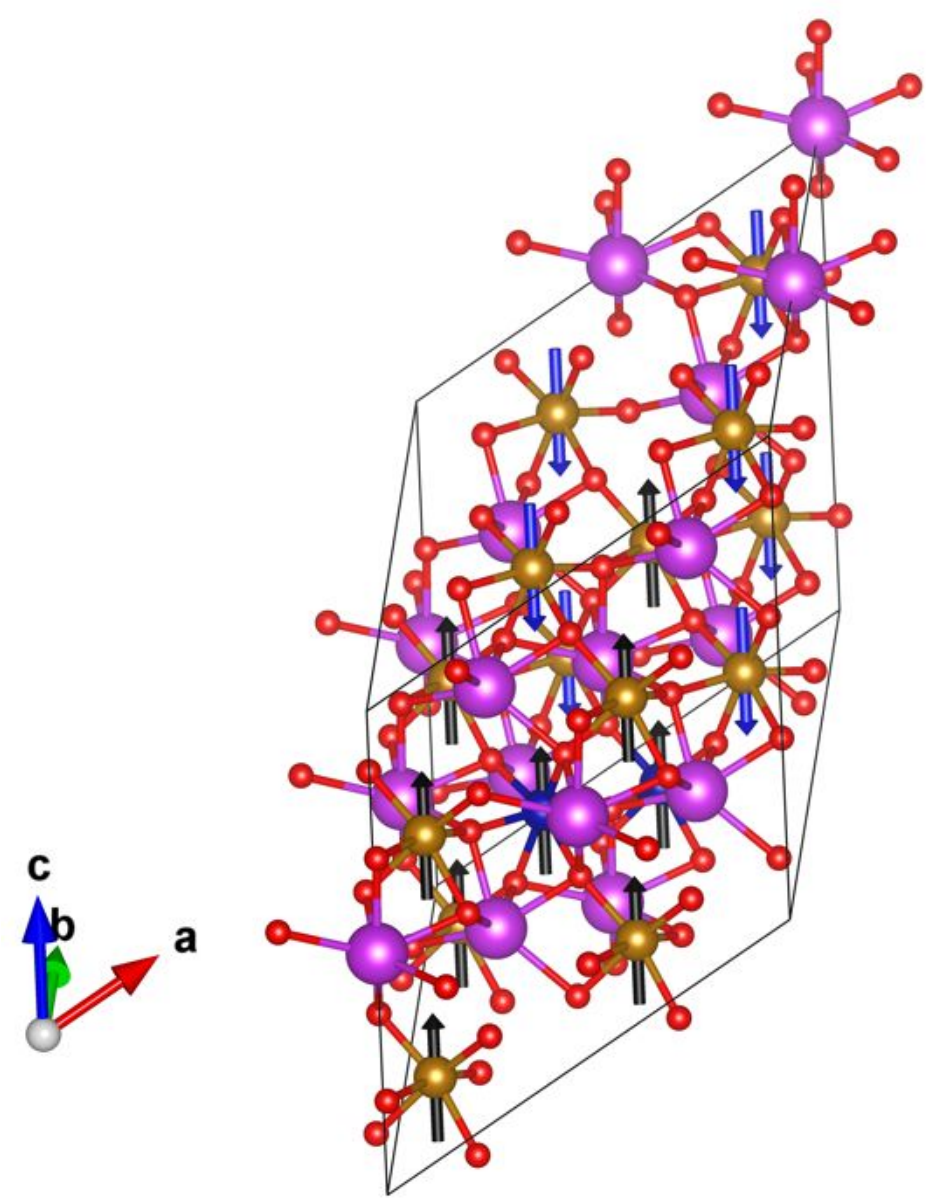


b)

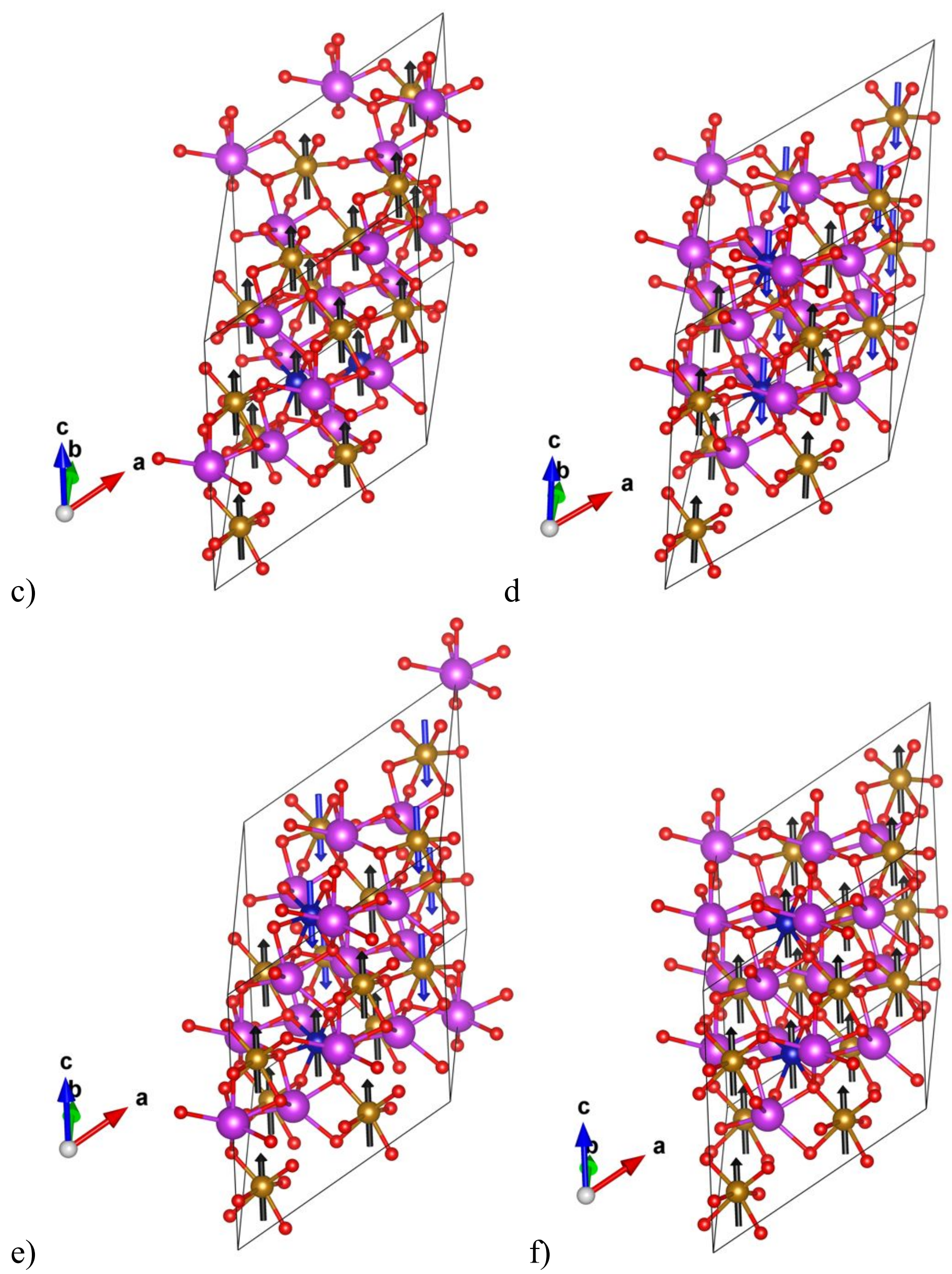
c)
d
e)
f)
c
b
a

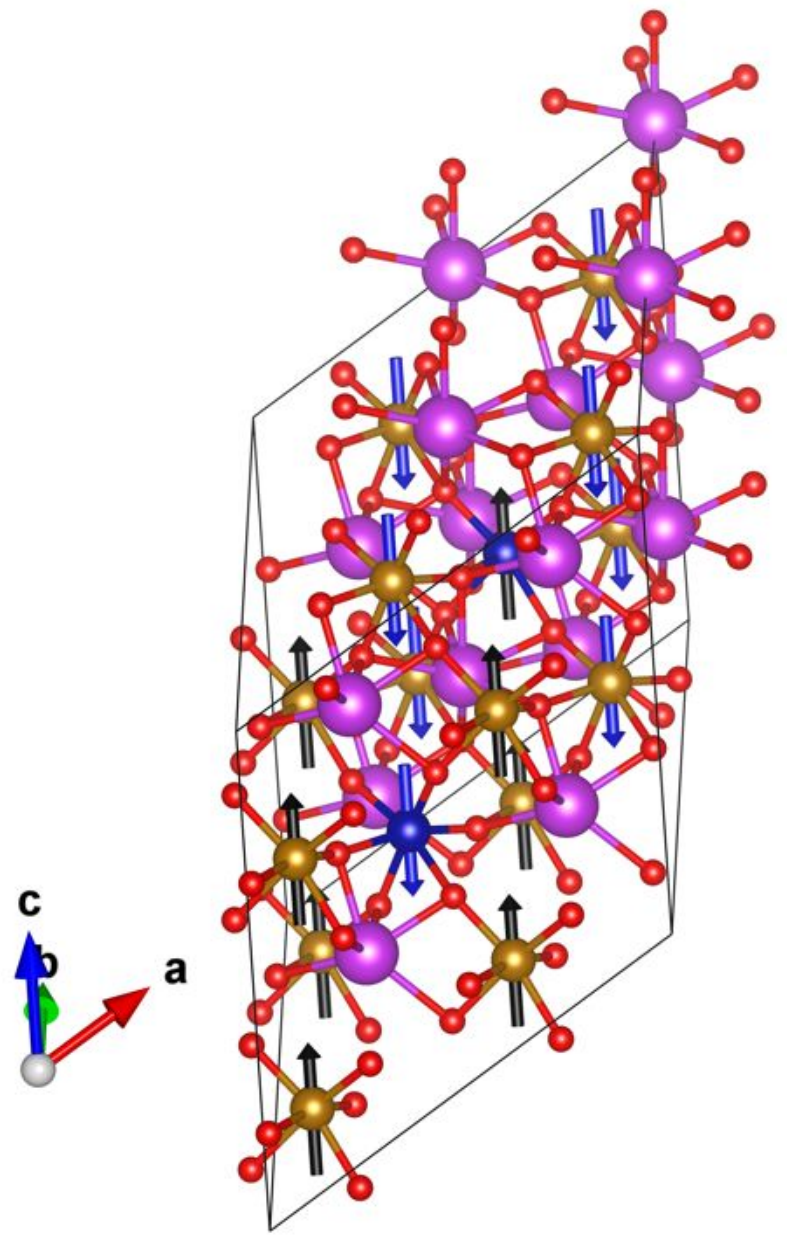


g)

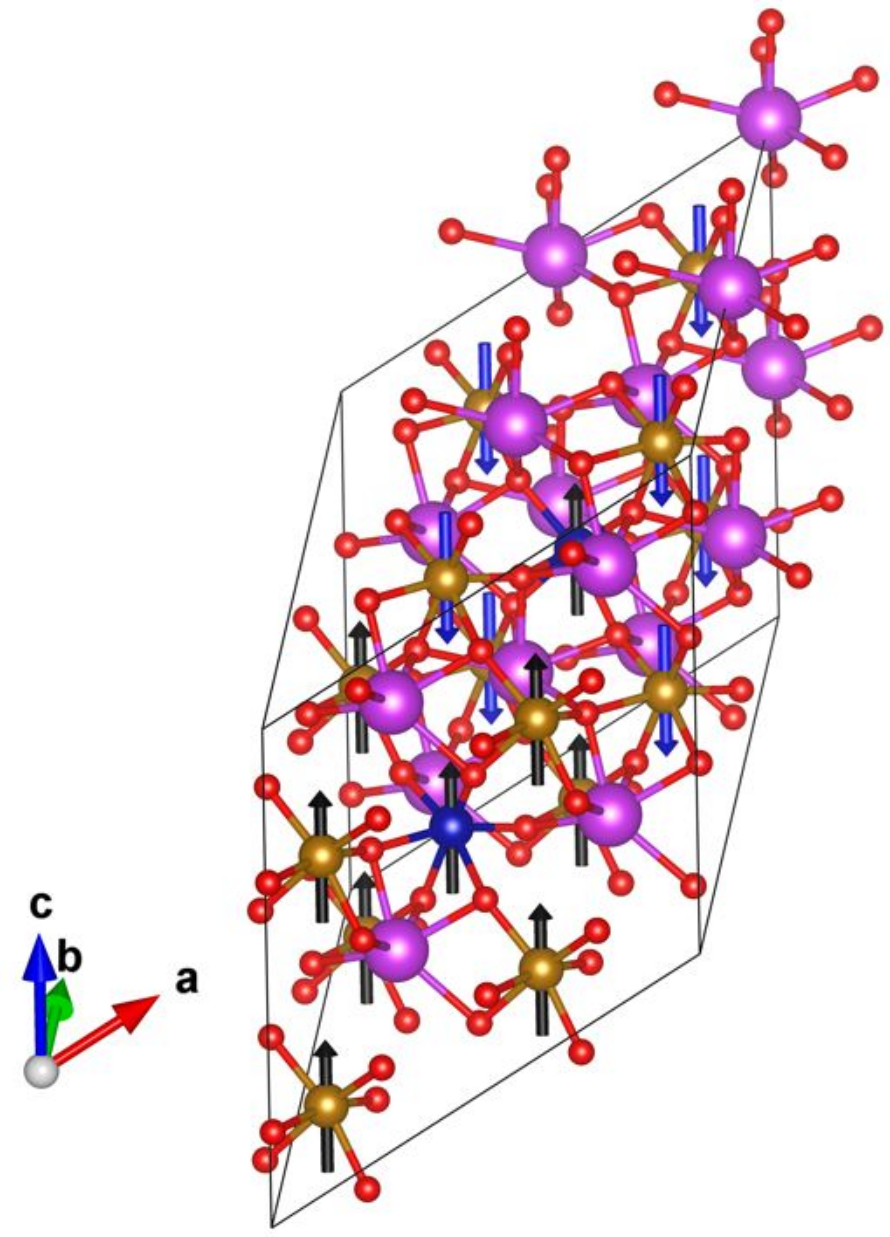


h)

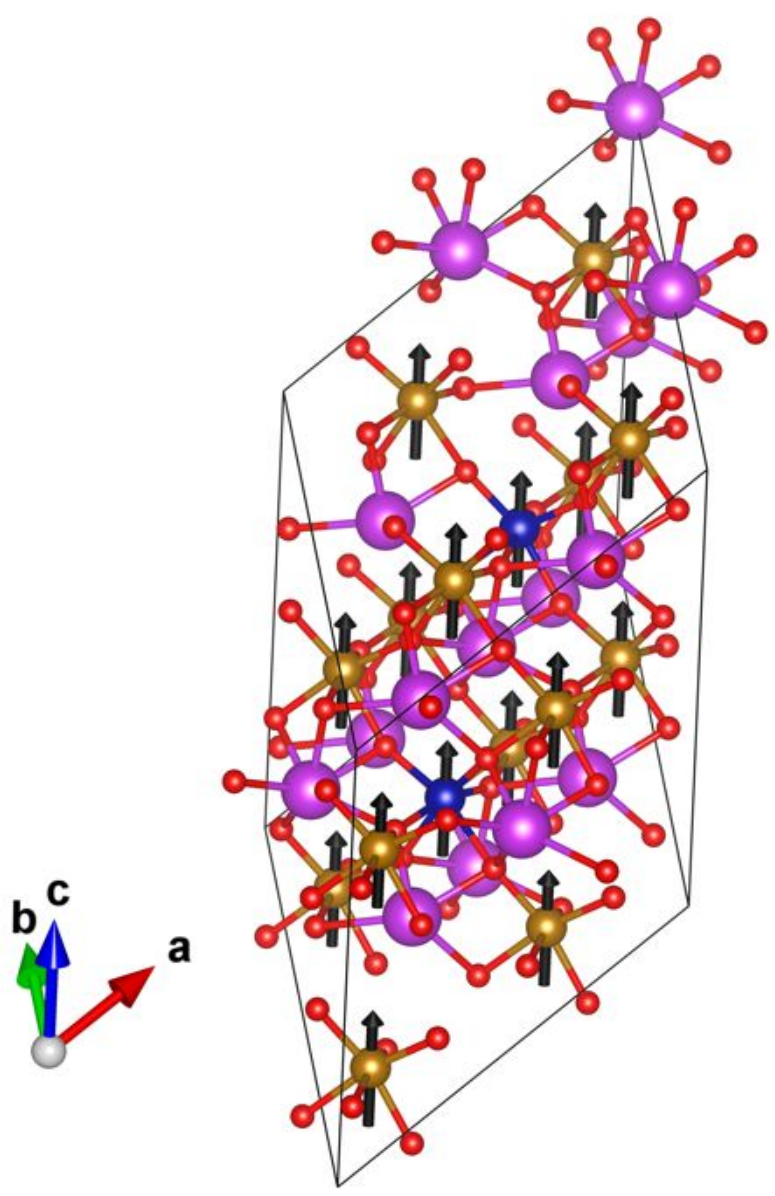


i)

Figure S1. Supercell of a) Conf. 1, b) Conf. 2, c) Conf. 3, d) Conf. 4, e) Conf. 5, f) Conf. 6, g) Conf. 7, h) Conf. 8, i) Conf. 9 of the $BiFe_{0.875}Co_{0.125}O_3$ solid solution.

Table S1. Magnetic moments ($\mu_B$) of all ions obtained from the DOS calculations of pristine $BiFeO_3$ and nine distinct configurations the $BiFe_{0.875}Co_{0.125}O_3$ solid solution.

| printine $BiFeO_3$ | | | | | Conf. 1 | | | | | Conf. 2 | | | | | Conf. 3 | | | | |
|---|---|---|---|---|---|---|---|---|---|---|---|---|---|---|---|---|---|---|---|
| magnetization (x) | | | | | magnetization (x) | | | | | magnetization (x) | | | | | magnetization (x) | | | | |
| # of ion | s | p | d | tot | # of ion | s | p | d | tot | # of ion | s | p | d | tot | # of ion | s | p | d | tot |
| 1. Bi | 0.000 | -0.003 | 0.001 | -0.002 | 1 | 0.000 | -0.004 | 0.001 | -0.002 | 1 | 0.001 | -0.004 | 0.001 | -0.002 | 1 | 0.004 | 0.012 | 0.001 | 0.017 |
| 2. Bi | 0.000 | -0.003 | 0.001 | -0.002 | 2 | 0.001 | -0.004 | 0.001 | -0.002 | 2 | 0.001 | -0.004 | 0.001 | -0.002 | 2 | 0.002 | 0.012 | 0.001 | 0.015 |
| 3. Bi | 0.000 | -0.003 | 0.001 | -0.002 | 3 | 0.001 | -0.005 | 0.001 | -0.003 | 3 | 0.001 | -0.005 | 0.001 | -0.003 | 3 | 0.004 | 0.013 | 0.001 | 0.018 |
| 4. Bi | 0.000 | -0.003 | 0.001 | -0.002 | 4 | -0.000 | 0.001 | 0.001 | 0.001 | 4 | 0.002 | -0.001 | 0.001 | 0.002 | 4 | 0.004 | 0.010 | 0.001 | 0.015 |
| 5. Bi | 0.000 | -0.003 | 0.001 | -0.002 | 5 | 0.001 | -0.004 | 0.001 | -0.002 | 5 | 0.001 | -0.004 | 0.001 | -0.003 | 5 | 0.004 | 0.010 | 0.001 | 0.015 |

| | | | | | | | | | | | | | | | | | | | |
|---|---|---|---|---|---|---|---|---|---|---|---|---|---|---|---|---|---|---|---|
| 6. Bi | 0.000 | -0.003 | 0.001 | -0.002 | 6 | -0.000 | -0.004 | 0.001 | -0.003 | 6 | 0.002 | -0.003 | 0.001 | 0.000 | 6 | 0.004 | 0.006 | 0.001 | 0.011 |
| 7. Bi | 0.000 | -0.003 | 0.001 | -0.002 | 7 | -0.000 | -0.004 | 0.001 | -0.004 | 7 | 0.003 | -0.000 | 0.001 | 0.004 | 7 | 0.005 | 0.016 | 0.002 | 0.023 |
| 8. Bi | 0.000 | -0.003 | 0.001 | -0.002 | 8 | 0.002 | -0.004 | 0.001 | -0.001 | 8 | 0.002 | -0.004 | 0.001 | -0.001 | 8 | 0.004 | 0.012 | 0.001 | 0.017 |
| 9. Bi | -0.000 | 0.003 | -0.001 | 0.002 | 9 | -0.000 | 0.006 | -0.001 | 0.005 | 9 | 0.002 | 0.002 | -0.001 | 0.002 | 9 | 0.004 | 0.010 | 0.001 | 0.015 |
| 10. Bi | -0.000 | 0.003 | -0.001 | 0.002 | 10 | -0.002 | 0.004 | -0.001 | 0.001 | 10 | 0.001 | 0.004 | -0.000 | 0.005 | 10 | 0.005 | 0.010 | 0.001 | 0.016 |
| 11. Bi | -0.000 | 0.003 | -0.001 | 0.002 | 11 | -0.000 | 0.005 | -0.001 | 0.003 | 11 | 0.002 | 0.005 | -0.000 | 0.007 | 11 | 0.004 | 0.013 | 0.001 | 0.018 |
| 12. Bi | -0.000 | 0.003 | -0.001 | 0.002 | 12 | -0.000 | 0.004 | -0.001 | 0.003 | 12 | -0.000 | 0.003 | -0.001 | 0.002 | 12 | 0.004 | 0.012 | 0.001 | 0.017 |
| 13. Bi | -0.000 | 0.003 | -0.001 | 0.002 | 13 | -0.000 | -0.000 | -0.001 | -0.002 | 13 | 0.003 | 0.001 | 0.000 | 0.004 | 13 | 0.006 | 0.013 | 0.002 | 0.021 |
| 14. Bi | -0.000 | 0.003 | -0.001 | 0.002 | 14 | -0.001 | 0.003 | -0.001 | 0.002 | 14 | -0.001 | 0.004 | -0.001 | 0.002 | 14 | 0.004 | 0.012 | 0.001 | 0.017 |
| 15. Bi | -0.000 | 0.003 | -0.001 | 0.002 | 15 | -0.001 | 0.004 | -0.001 | 0.002 | 15 | -0.001 | 0.003 | -0.001 | 0.002 | 15 | 0.005 | 0.011 | 0.001 | 0.017 |
| 16. Bi | -0.000 | 0.003 | -0.001 | 0.002 | 16 | -0.000 | 0.001 | -0.001 | 0.000 | 16 | 0.000 | 0.003 | -0.001 | 0.002 | 16 | 0.004 | 0.012 | 0.001 | 0.016 |
| 17. Fe | 0.017 | 0.015 | 4.344 | 4.377 | 17 | 0.012 | 0.013 | 4.219 | 4.244 | 17 | 0.012 | 0.013 | 4.219 | 4.244 | 17 | 0.026 | 0.040 | 4.291 | 4.357 |
| 18. Fe | 0.017 | 0.015 | 4.344 | 4.377 | 18 | 0.012 | 0.013 | 4.215 | 4.240 | 18 | 0.014 | 0.017 | 4.231 | 4.262 | 18 | 0.026 | 0.040 | 4.289 | 4.356 |
| 19. Fe | 0.017 | 0.015 | 4.344 | 4.377 | 19 | 0.012 | 0.012 | 4.214 | 4.238 | 19 | 0.014 | 0.017 | 4.231 | 4.262 | 19 | 0.026 | 0.041 | 4.293 | 4.359 |
| 20. Fe | 0.017 | 0.015 | 4.344 | 4.377 | 20 | 0.012 | 0.013 | 4.215 | 4.240 | 20 | 0.013 | 0.018 | 4.228 | 4.259 | 20 | 0.026 | 0.040 | 4.291 | 4.357 |
| 21. Fe | 0.017 | 0.015 | 4.344 | 4.377 | 21 | 0.012 | 0.013 | 4.217 | 4.242 | 21 | 0.014 | 0.018 | 4.231 | 4.264 | 21 | 0.026 | 0.040 | 4.293 | 4.358 |
| 22. Fe | 0.017 | 0.015 | 4.344 | 4.377 | 22 | 0.012 | 0.014 | 4.211 | 4.236 | 22 | 0.014 | 0.018 | 4.230 | 4.263 | 22 | 0.026 | 0.040 | 4.293 | 4.358 |
| 23. Fe | 0.017 | 0.015 | 4.344 | 4.377 | 23 | 0.012 | 0.014 | 4.218 | 4.243 | 23 | 0.011 | 0.014 | 4.217 | 4.242 | 23 | 0.026 | 0.041 | 4.292 | 4.358 |
| 24. Fe | 0.017 | 0.015 | 4.344 | 4.377 | 24 | -0.012 | -0.014 | -4.218 | -4.243 | 24 | -0.012 | -0.013 | -4.218 | -4.243 | 24 | 0.026 | 0.040 | 4.298 | 4.364 |
| 25. Fe | -0.017 | -0.015 | -4.344 | -4.377 | 25 | -0.012 | -0.014 | -4.210 | -4.236 | 25 | -0.012 | -0.014 | -4.209 | -4.235 | 25 | 0.026 | 0.040 | 4.264 | 4.330 |
| 26. Fe | -0.017 | -0.015 | -4.344 | -4.377 | 26 | -0.012 | -0.013 | -4.216 | -4.241 | 26 | -0.012 | -0.013 | -4.215 | -4.240 | 26 | 0.026 | 0.038 | 4.268 | 4.331 |
| 27. Fe | -0.017 | -0.015 | -4.344 | -4.377 | 27 | -0.012 | -0.013 | -4.216 | -4.241 | 27 | -0.012 | -0.013 | -4.214 | -4.239 | 27 | 0.026 | 0.040 | 4.260 | 4.326 |
| 28. Fe | -0.017 | -0.015 | -4.344 | -4.377 | 28 | -0.012 | -0.012 | -4.214 | -4.238 | 28 | -0.011 | -0.011 | -4.211 | -4.234 | 28 | 0.026 | 0.037 | 4.261 | 4.324 |
| 29. Fe | -0.017 | -0.015 | -4.344 | -4.377 | 29 | -0.012 | -0.013 | -4.219 | -4.244 | 29 | -0.012 | -0.013 | -4.217 | -4.241 | 29 | 0.026 | 0.040 | 4.297 | 4.363 |
| 30. Fe | -0.017 | -0.015 | -4.344 | -4.377 | 30 | -0.012 | -0.013 | -4.216 | -4.241 | 30 | -0.012 | -0.013 | -4.211 | -4.236 | 30 | 0.026 | 0.039 | 4.285 | 4.350 |
| 31. Fe | -0.017 | -0.015 | -4.344 | -4.377 | 31 | -0.003 | -0.003 | -3.082 | -3.088 | 31 | 0.016 | 0.028 | 3.150 | 3.194 | 31 | 0.018 | 0.030 | 3.145 | 3.193 |
| 32. Fe | -0.017 | -0.015 | -4.344 | -4.377 | 32 | 0.003 | 0.003 | 3.082 | 3.088 | 32 | 0.005 | 0.010 | 3.088 | 3.104 | 32 | 0.024 | 0.030 | 2.459 | 2.512 |
| 33. O | 0.001 | 0.032 | 0.000 | 0.033 | 33 | 0.001 | 0.024 | 0.000 | 0.024 | 33 | 0.009 | 0.163 | 0.000 | 0.172 | 33 | 0.010 | 0.181 | 0.000 | 0.192 |
| 34. O | 0.001 | 0.032 | 0.000 | 0.033 | 34 | 0.001 | 0.030 | 0.000 | 0.031 | 34 | 0.001 | 0.037 | 0.000 | 0.038 | 34 | 0.010 | 0.170 | 0.000 | 0.180 |
| 35. O | 0.001 | 0.032 | 0.000 | 0.033 | 35 | 0.001 | 0.032 | 0.000 | 0.033 | 35 | 0.001 | 0.034 | 0.000 | 0.035 | 35 | 0.010 | 0.145 | 0.000 | 0.155 |
| 36. O | 0.001 | 0.032 | 0.000 | 0.033 | 36 | 0.001 | 0.034 | 0.000 | 0.035 | 36 | 0.001 | 0.036 | 0.000 | 0.037 | 36 | 0.010 | 0.153 | 0.000 | 0.162 |
| 37. O | 0.001 | 0.032 | 0.000 | 0.033 | 37 | 0.001 | 0.035 | 0.000 | 0.036 | 37 | 0.001 | 0.042 | 0.000 | 0.043 | 37 | 0.010 | 0.160 | 0.000 | 0.170 |
| 38. O | 0.001 | 0.032 | 0.000 | 0.033 | 38 | 0.001 | 0.031 | 0.000 | 0.032 | 38 | 0.001 | 0.042 | 0.000 | 0.043 | 38 | 0.010 | 0.155 | 0.000 | 0.165 |
| 39. O | 0.001 | 0.032 | 0.000 | 0.033 | 39 | 0.001 | 0.029 | 0.000 | 0.030 | 39 | 0.001 | 0.036 | 0.000 | 0.037 | 39 | 0.010 | 0.169 | 0.000 | 0.179 |
| 40. O | 0.001 | 0.032 | 0.000 | 0.033 | 40 | 0.001 | 0.030 | 0.000 | 0.031 | 40 | 0.001 | 0.047 | 0.000 | 0.048 | 40 | 0.009 | 0.115 | 0.000 | 0.124 |
| 41. O | 0.001 | 0.032 | 0.000 | 0.033 | 41 | 0.001 | 0.029 | 0.000 | 0.030 | 41 | 0.009 | 0.148 | 0.000 | 0.157 | 41 | 0.010 | 0.163 | 0.000 | 0.173 |
| 42. O | 0.001 | 0.032 | 0.000 | 0.033 | 42 | 0.001 | 0.035 | 0.000 | 0.036 | 42 | 0.001 | 0.036 | 0.000 | 0.037 | 42 | 0.010 | 0.166 | 0.000 | 0.176 |
| 43. O | 0.001 | 0.032 | 0.000 | 0.033 | 43 | 0.002 | 0.114 | 0.000 | 0.116 | 43 | 0.002 | 0.131 | 0.000 | 0.133 | 43 | 0.007 | 0.003 | 0.000 | 0.010 |
| 44. O | 0.001 | 0.032 | 0.000 | 0.033 | 44 | 0.001 | 0.035 | 0.000 | 0.035 | 44 | 0.001 | 0.038 | 0.000 | 0.039 | 44 | 0.010 | 0.162 | 0.000 | 0.172 |
| 45. O | 0.001 | 0.032 | 0.000 | 0.033 | 45 | 0.001 | 0.035 | 0.000 | 0.036 | 45 | 0.001 | 0.037 | 0.000 | 0.038 | 45 | 0.010 | 0.146 | 0.000 | 0.156 |
| 46. O | 0.001 | 0.032 | 0.000 | 0.033 | 46 | 0.001 | 0.031 | 0.000 | 0.032 | 46 | 0.001 | 0.037 | 0.000 | 0.038 | 46 | 0.010 | 0.162 | 0.000 | 0.172 |
| 47. O | 0.001 | 0.032 | 0.000 | 0.033 | 47 | 0.001 | 0.033 | 0.000 | 0.034 | 47 | 0.001 | 0.042 | 0.000 | 0.043 | 47 | 0.010 | 0.166 | 0.000 | 0.176 |
| 48. O | 0.001 | 0.032 | 0.000 | 0.033 | 48 | 0.001 | 0.034 | 0.000 | 0.035 | 48 | 0.001 | 0.041 | 0.000 | 0.041 | 48 | 0.010 | 0.163 | 0.000 | 0.173 |
| 49. O | 0.001 | 0.032 | 0.000 | 0.033 | 49 | 0.001 | 0.005 | 0.000 | 0.006 | 49 | 0.009 | 0.149 | 0.000 | 0.158 | 49 | 0.010 | 0.168 | 0.000 | 0.178 |
| 50. O | 0.001 | 0.032 | 0.000 | 0.033 | 50 | 0.001 | 0.035 | 0.000 | 0.036 | 50 | 0.001 | 0.038 | 0.000 | 0.039 | 50 | 0.010 | 0.165 | 0.000 | 0.175 |
| 51. O | 0.001 | 0.032 | 0.000 | 0.033 | 51 | 0.001 | 0.033 | 0.000 | 0.034 | 51 | 0.001 | 0.034 | 0.000 | 0.035 | 51 | 0.010 | 0.164 | 0.000 | 0.174 |
| 52. O | 0.001 | 0.032 | 0.000 | 0.033 | 52 | 0.001 | 0.034 | 0.000 | 0.035 | 52 | 0.001 | 0.042 | 0.000 | 0.043 | 52 | 0.010 | 0.165 | 0.000 | 0.175 |
| 53. O | 0.001 | 0.032 | 0.000 | 0.033 | 53 | 0.001 | 0.042 | 0.000 | 0.043 | 53 | 0.001 | 0.048 | 0.000 | 0.050 | 53 | 0.007 | 0.002 | 0.000 | 0.009 |
| 54. O | 0.001 | 0.032 | 0.000 | 0.033 | 54 | 0.001 | 0.034 | 0.000 | 0.035 | 54 | 0.001 | 0.038 | 0.000 | 0.038 | 54 | 0.010 | 0.157 | 0.000 | 0.167 |
| 55. O | 0.001 | 0.032 | 0.000 | 0.033 | 55 | 0.001 | 0.032 | 0.000 | 0.033 | 55 | 0.001 | 0.036 | 0.000 | 0.037 | 55 | 0.010 | 0.163 | 0.000 | 0.173 |
| 56. O | 0.001 | 0.032 | 0.000 | 0.033 | 56 | 0.001 | 0.043 | 0.000 | 0.044 | 56 | 0.001 | 0.043 | 0.000 | 0.044 | 56 | 0.010 | 0.153 | 0.000 | 0.163 |
| 57. O | -0.001 | -0.032 | 0.000 | -0.033 | 57 | -0.001 | -0.032 | 0.000 | -0.033 | 57 | -0.001 | -0.029 | 0.000 | -0.030 | 57 | 0.010 | 0.161 | 0.000 | 0.171 |
| 58. O | -0.001 | -0.032 | 0.000 | -0.033 | 58 | -0.001 | -0.036 | 0.000 | -0.036 | 58 | -0.000 | -0.026 | 0.000 | -0.026 | 58 | 0.010 | 0.166 | 0.000 | 0.176 |
| 59. O | -0.001 | -0.032 | 0.000 | -0.033 | 59 | -0.001 | -0.031 | 0.000 | -0.031 | 59 | -0.001 | -0.028 | 0.000 | -0.029 | 59 | 0.010 | 0.164 | 0.000 | 0.174 |
| 60. O | -0.001 | -0.032 | 0.000 | -0.033 | 60 | -0.001 | -0.031 | 0.000 | -0.031 | 60 | -0.001 | -0.026 | 0.000 | -0.026 | 60 | 0.010 | 0.151 | 0.000 | 0.160 |
| 61. O | -0.001 | -0.032 | 0.000 | -0.033 | 61 | -0.001 | -0.021 | 0.000 | -0.022 | 61 | 0.009 | 0.222 | 0.000 | 0.231 | 61 | 0.010 | 0.232 | 0.000 | 0.242 |
| 62. O | -0.001 | -0.032 | 0.000 | -0.033 | 62 | -0.001 | -0.030 | 0.000 | -0.030 | 62 | -0.001 | -0.031 | 0.000 | -0.032 | 62 | 0.010 | 0.167 | 0.000 | 0.177 |
| 63. O | -0.001 | -0.032 | 0.000 | -0.033 | 63 | -0.001 | -0.034 | 0.000 | -0.035 | 63 | -0.000 | -0.030 | 0.000 | -0.030 | 63 | 0.010 | 0.164 | 0.000 | 0.174 |
| 64. O | -0.001 | -0.032 | 0.000 | -0.033 | 64 | -0.001 | -0.031 | 0.000 | -0.032 | 64 | -0.000 | -0.025 | 0.000 | -0.025 | 64 | 0.010 | 0.168 | 0.000 | 0.178 |
| 65. O | -0.001 | -0.032 | 0.000 | -0.033 | 65 | -0.001 | -0.035 | 0.000 | -0.035 | 65 | -0.001 | -0.027 | 0.000 | -0.027 | 65 | 0.010 | 0.168 | 0.000 | 0.178 |
| 66. O | -0.001 | -0.032 | 0.000 | -0.033 | 66 | -0.002 | -0.117 | 0.000 | -0.118 | 66 | 0.009 | 0.147 | 0.000 | 0.156 | 66 | 0.010 | 0.169 | 0.000 | 0.179 |
| 67. O | -0.001 | -0.032 | 0.000 | -0.033 | 67 | -0.001 | -0.028 | 0.000 | -0.029 | 67 | -0.001 | -0.021 | 0.000 | -0.022 | 67 | 0.007 | 0.045 | 0.000 | 0.052 |
| 68. O | -0.001 | -0.032 | 0.000 | -0.033 | 68 | -0.001 | -0.035 | 0.000 | -0.036 | 68 | -0.001 | -0.034 | 0.000 | -0.035 | 68 | 0.010 | 0.155 | 0.000 | 0.165 |
| 69. O | -0.001 | -0.032 | 0.000 | -0.033 | 69 | -0.001 | -0.034 | 0.000 | -0.034 | 69 | -0.001 | -0.031 | 0.000 | -0.031 | 69 | 0.010 | 0.163 | 0.000 | 0.173 |
| 70. O | -0.001 | -0.032 | 0.000 | -0.033 | 70 | -0.001 | -0.035 | 0.000 | -0.035 | 70 | -0.000 | -0.026 | 0.000 | -0.027 | 70 | 0.010 | 0.157 | 0.000 | 0.167 |
| 71. O | -0.001 | -0.032 | 0.000 | -0.033 | 71 | -0.001 | -0.031 | 0.000 | -0.032 | 71 | -0.001 | -0.031 | 0.000 | -0.031 | 71 | 0.010 | 0.165 | 0.000 | 0.175 |
| 72. O | -0.001 | -0.032 | 0.000 | -0.033 | 72 | -0.001 | -0.036 | 0.000 | -0.037 | 72 | -0.000 | -0.027 | 0.000 | -0.027 | 72 | 0.010 | 0.167 | 0.000 | 0.178 |
| 73. O | -0.001 | -0.032 | 0.000 | -0.033 | 73 | -0.001 | -0.039 | 0.000 | -0.040 | 73 | -0.001 | -0.035 | 0.000 | -0.036 | 73 | 0.010 | 0.163 | 0.000 | 0.173 |
| 74. O | -0.001 | -0.032 | 0.000 | -0.033 | 74 | -0.001 | -0.034 | 0.000 | -0.035 | 74 | -0.001 | -0.032 | 0.000 | -0.032 | 74 | 0.010 | 0.165 | 0.000 | 0.175 |
| 75. O | -0.001 | -0.032 | 0.000 | -0.033 | 75 | -0.001 | -0.044 | 0.000 | -0.045 | 75 | 0.009 | 0.146 | 0.000 | 0.155 | 75 | 0.010 | 0.165 | 0.000 | 0.175 |
| 76. O | -0.001 | -0.032 | 0.000 | -0.033 | 76 | -0.001 | -0.035 | 0.000 | -0.035 | 76 | -0.000 | -0.028 | 0.000 | -0.028 | 76 | 0.010 | 0.164 | 0.000 | 0.175 |
| 77. O | -0.001 | -0.032 | 0.000 | -0.033 | 77 | -0.001 | -0.032 | 0.000 | -0.033 | 77 | -0.001 | -0.025 | 0.000 | -0.026 | 77 | 0.010 | 0.166 | 0.000 | 0.176 |
| 78. O | -0.001 | -0.032 | 0.000 | -0.033 | 78 | -0.001 | -0.033 | 0.000 | -0.033 | 78 | -0.001 | -0.028 | 0.000 | -0.029 | 78 | 0.010 | 0.159 | 0.000 | 0.169 |
| 79. O | -0.001 | -0.032 | 0.000 | -0.033 | 79 | -0.001 | -0.034 | 0.000 | -0.034 | 79 | -0.001 | -0.034 | 0.000 | -0.035 | 79 | 0.010 | 0.162 | 0.000 | 0.172 |
| 80. O | -0.001 | -0.032 | 0.000 | -0.033 | 80 | -0.001 | -0.009 | 0.000 | -0.010 | 80 | 0.000 | 0.012 | 0.000 | 0.012 | 80 | 0.007 | 0.039 | 0.000 | 0.047 |
| tot | 0.000 | 0.000 | 0.000 | 0.000 | tot | -0.001 | -0.005 | -0.001 | -0.006 | tot | 0.116 | 1.392 | 6.334 | 7.842 | tot | 0.941 | 8.063 | 65.595 | 74.599 |

| Conf. 4 | Conf. 5 | Conf. 6 |
|---|---|---|
| magnetization (x) | magnetization (x) | magnetization (x) |
| # of ion s p d tot | # of ion s p d tot | # of ion s p d tot |

| | | | | |
|---|---|---|---|---|
| 1 | 0.000 | -0.003 | 0.001 | -0.002 |
| 2 | 0.000 | -0.003 | 0.001 | -0.002 |
| 3 | -0.001 | -0.001 | 0.001 | -0.001 |
| 4 | -0.001 | -0.001 | 0.001 | -0.001 |
| 5 | -0.001 | 0.001 | 0.001 | 0.001 |
| 6 | -0.001 | 0.001 | 0.001 | 0.001 |
| 7 | -0.000 | -0.003 | 0.001 | -0.003 |
| 8 | -0.000 | -0.003 | 0.001 | -0.003 |
| 9 | -0.001 | 0.008 | -0.001 | 0.006 |
| 10 | -0.001 | 0.008 | -0.001 | 0.006 |
| 11 | -0.002 | 0.005 | -0.001 | 0.002 |
| 12 | -0.002 | 0.005 | -0.001 | 0.002 |
| 13 | -0.002 | 0.005 | -0.001 | 0.002 |
| 14 | -0.002 | 0.005 | -0.001 | 0.002 |
| 15 | -0.001 | 0.004 | -0.001 | 0.002 |
| 16 | -0.001 | 0.004 | -0.001 | 0.002 |
| 17 | 0.012 | 0.012 | 4.214 | 4.238 |
| 18 | 0.012 | 0.012 | 4.214 | 4.238 |
| 19 | 0.012 | 0.013 | 4.214 | 4.240 |
| 20 | 0.012 | 0.013 | 4.214 | 4.240 |
| 21 | 0.012 | 0.013 | 4.213 | 4.238 |
| 22 | 0.012 | 0.013 | 4.213 | 4.238 |
| 23 | 0.012 | 0.014 | 4.209 | 4.235 |
| 24 | 0.012 | 0.014 | 4.209 | 4.235 |
| 25 | -0.012 | -0.014 | -4.219 | -4.244 |
| 26 | -0.012 | -0.014 | -4.219 | -4.244 |
| 27 | -0.011 | -0.013 | -4.218 | -4.243 |
| 28 | -0.011 | -0.013 | -4.218 | -4.243 |
| 29 | -0.012 | -0.013 | -4.219 | -4.243 |
| 30 | -0.012 | -0.013 | -4.219 | -4.243 |
| 31 | -0.003 | -0.003 | -3.084 | -3.090 |
| 32 | -0.003 | -0.003 | -3.084 | -3.090 |
| 33 | 0.001 | 0.003 | 0.000 | 0.003 |
| 34 | 0.001 | 0.003 | 0.000 | 0.003 |
| 35 | 0.001 | 0.030 | 0.000 | 0.030 |
| 36 | 0.001 | 0.030 | 0.000 | 0.030 |
| 37 | 0.001 | 0.032 | 0.000 | 0.033 |
| 38 | 0.001 | 0.032 | 0.000 | 0.033 |
| 39 | 0.001 | 0.038 | 0.000 | 0.039 |
| 40 | 0.001 | 0.038 | 0.000 | 0.039 |
| 41 | 0.001 | 0.021 | 0.000 | 0.022 |
| 42 | 0.001 | 0.021 | 0.000 | 0.022 |
| 43 | 0.001 | 0.029 | 0.000 | 0.029 |
| 44 | 0.001 | 0.029 | 0.000 | 0.029 |
| 45 | 0.001 | 0.029 | 0.000 | 0.030 |
| 46 | 0.001 | 0.029 | 0.000 | 0.030 |
| 47 | 0.001 | 0.028 | 0.000 | 0.029 |
| 48 | 0.001 | 0.028 | 0.000 | 0.029 |
| 49 | 0.001 | 0.025 | 0.000 | 0.026 |
| 50 | 0.001 | 0.025 | 0.000 | 0.026 |
| 51 | 0.001 | 0.030 | 0.000 | 0.030 |
| 52 | 0.001 | 0.030 | 0.000 | 0.030 |
| 53 | 0.001 | 0.032 | 0.000 | 0.033 |
| 54 | 0.001 | 0.032 | 0.000 | 0.033 |
| 55 | 0.001 | 0.030 | 0.000 | 0.031 |
| 56 | 0.001 | 0.030 | 0.000 | 0.031 |
| 57 | -0.001 | -0.038 | 0.000 | -0.039 |
| 58 | -0.001 | -0.038 | 0.000 | -0.039 |
| 59 | -0.001 | -0.035 | 0.000 | -0.035 |
| 60 | -0.001 | -0.035 | 0.000 | -0.035 |
| 61 | -0.002 | -0.122 | 0.000 | -0.124 |
| 62 | -0.002 | -0.122 | 0.000 | -0.124 |
| 63 | -0.001 | -0.040 | 0.000 | -0.041 |
| 64 | -0.001 | -0.040 | 0.000 | -0.041 |
| 65 | -0.001 | -0.042 | 0.000 | -0.043 |
| 66 | -0.001 | -0.042 | 0.000 | -0.043 |
| 67 | -0.001 | -0.034 | 0.000 | -0.035 |
| 68 | -0.001 | -0.034 | 0.000 | -0.035 |
| 69 | -0.001 | -0.037 | 0.000 | -0.038 |
| 70 | -0.001 | -0.037 | 0.000 | -0.038 |
| 71 | -0.001 | -0.038 | 0.000 | -0.039 |
| 72 | -0.001 | -0.038 | 0.000 | -0.039 |
| 73 | -0.001 | -0.035 | 0.000 | -0.036 |
| 74 | -0.001 | -0.035 | 0.000 | -0.036 |
| 75 | -0.001 | -0.033 | 0.000 | -0.033 |
| 76 | -0.001 | -0.033 | 0.000 | -0.033 |
| 77 | -0.001 | -0.036 | 0.000 | -0.037 |
| 78 | -0.001 | -0.036 | 0.000 | -0.037 |
| 79 | -0.001 | -0.033 | 0.000 | -0.034 |
| 80 | -0.001 | -0.033 | 0.000 | -0.034 |
| tot | -0.003 | -0.337 | 2.219 | 1.879 |

| | | | | |
|---|---|---|---|---|
| 1 | 0.000 | -0.004 | 0.001 | -0.002 |
| 2 | 0.000 | -0.003 | 0.001 | -0.002 |
| 3 | 0.000 | -0.003 | 0.001 | -0.002 |
| 4 | 0.002 | -0.001 | 0.001 | 0.002 |
| 5 | -0.001 | -0.001 | 0.001 | -0.000 |
| 6 | 0.002 | -0.003 | 0.001 | -0.000 |
| 7 | 0.002 | 0.002 | 0.001 | 0.005 |
| 8 | -0.000 | 0.001 | 0.001 | 0.002 |
| 9 | 0.000 | 0.003 | -0.001 | 0.002 |
| 10 | 0.001 | 0.007 | -0.000 | 0.008 |
| 11 | 0.001 | 0.005 | -0.000 | 0.006 |
| 12 | -0.002 | 0.004 | -0.001 | 0.001 |
| 13 | 0.002 | 0.005 | 0.000 | 0.007 |
| 14 | -0.001 | 0.005 | -0.001 | 0.003 |
| 15 | -0.001 | 0.004 | -0.001 | 0.002 |
| 16 | -0.000 | 0.006 | -0.001 | 0.005 |
| 17 | 0.012 | 0.013 | 4.216 | 4.241 |
| 18 | 0.014 | 0.017 | 4.231 | 4.263 |
| 19 | 0.014 | 0.018 | 4.221 | 4.254 |
| 20 | 0.013 | 0.018 | 4.226 | 4.257 |
| 21 | 0.015 | 0.018 | 4.229 | 4.261 |
| 22 | 0.014 | 0.017 | 4.225 | 4.256 |
| 23 | 0.013 | 0.021 | 4.225 | 4.259 |
| 24 | 0.012 | 0.013 | 4.215 | 4.240 |
| 25 | -0.012 | -0.013 | -4.218 | -4.243 |
| 26 | -0.012 | -0.013 | -4.216 | -4.240 |
| 27 | -0.012 | -0.013 | -4.217 | -4.242 |
| 28 | -0.012 | -0.012 | -4.217 | -4.241 |
| 29 | -0.012 | -0.013 | -4.217 | -4.241 |
| 30 | -0.011 | -0.012 | -4.214 | -4.238 |
| 31 | 0.016 | 0.028 | 3.150 | 3.194 |
| 32 | -0.003 | -0.002 | -3.083 | -3.089 |
| 33 | 0.009 | 0.159 | 0.000 | 0.168 |
| 34 | 0.001 | 0.031 | 0.000 | 0.032 |
| 35 | 0.001 | 0.035 | 0.000 | 0.036 |
| 36 | 0.001 | 0.036 | 0.000 | 0.037 |
| 37 | 0.001 | 0.037 | 0.000 | 0.038 |
| 38 | 0.001 | 0.039 | 0.000 | 0.040 |
| 39 | 0.001 | 0.033 | 0.000 | 0.034 |
| 40 | 0.001 | 0.051 | 0.000 | 0.052 |
| 41 | 0.009 | 0.143 | 0.000 | 0.152 |
| 42 | 0.001 | 0.006 | 0.000 | 0.006 |
| 43 | 0.001 | 0.040 | 0.000 | 0.040 |
| 44 | 0.001 | 0.035 | 0.000 | 0.036 |
| 45 | 0.001 | 0.033 | 0.000 | 0.034 |
| 46 | 0.001 | 0.037 | 0.000 | 0.038 |
| 47 | 0.001 | 0.046 | 0.000 | 0.047 |
| 48 | 0.001 | 0.039 | 0.000 | 0.040 |
| 49 | 0.009 | 0.146 | 0.000 | 0.155 |
| 50 | 0.001 | 0.028 | 0.000 | 0.028 |
| 51 | 0.001 | 0.033 | 0.000 | 0.034 |
| 52 | 0.001 | 0.035 | 0.000 | 0.036 |
| 53 | 0.001 | 0.037 | 0.000 | 0.037 |
| 54 | 0.001 | 0.034 | 0.000 | 0.035 |
| 55 | 0.001 | 0.033 | 0.000 | 0.034 |
| 56 | 0.001 | 0.041 | 0.000 | 0.042 |
| 57 | -0.001 | -0.031 | 0.000 | -0.031 |
| 58 | -0.001 | -0.026 | 0.000 | -0.026 |
| 59 | -0.001 | -0.033 | 0.000 | -0.034 |
| 60 | -0.001 | -0.029 | 0.000 | -0.029 |
| 61 | 0.009 | 0.216 | 0.000 | 0.225 |
| 62 | -0.001 | -0.043 | 0.000 | -0.044 |
| 63 | -0.000 | -0.033 | 0.000 | -0.034 |
| 64 | -0.000 | -0.028 | 0.000 | -0.028 |
| 65 | -0.001 | -0.027 | 0.000 | -0.028 |
| 66 | 0.009 | 0.147 | 0.000 | 0.156 |
| 67 | -0.000 | -0.021 | 0.000 | -0.022 |
| 68 | -0.001 | -0.036 | 0.000 | -0.036 |
| 69 | -0.001 | -0.033 | 0.000 | -0.034 |
| 70 | -0.000 | -0.028 | 0.000 | -0.029 |
| 71 | -0.001 | -0.030 | 0.000 | -0.031 |
| 72 | -0.000 | -0.026 | 0.000 | -0.026 |
| 73 | -0.001 | -0.030 | 0.000 | -0.030 |
| 74 | -0.001 | -0.036 | 0.000 | -0.036 |
| 75 | 0.009 | 0.143 | 0.000 | 0.152 |
| 76 | -0.001 | -0.112 | 0.000 | -0.114 |
| 77 | -0.001 | -0.025 | 0.000 | -0.026 |
| 78 | -0.001 | -0.032 | 0.000 | -0.032 |
| 79 | -0.001 | -0.037 | 0.000 | -0.037 |
| 80 | -0.000 | -0.020 | 0.000 | -0.020 |
| tot | 0.117 | 1.090 | 8.559 | 9.766 |

| | | | | |
|---|---|---|---|---|
| 1 | 0.004 | 0.011 | 0.001 | 0.017 |
| 2 | 0.004 | 0.011 | 0.001 | 0.017 |
| 3 | 0.004 | 0.007 | 0.001 | 0.012 |
| 4 | 0.004 | 0.007 | 0.001 | 0.012 |
| 5 | 0.005 | 0.009 | 0.001 | 0.015 |
| 6 | 0.005 | 0.009 | 0.001 | 0.015 |
| 7 | 0.004 | 0.011 | 0.001 | 0.017 |
| 8 | 0.004 | 0.011 | 0.001 | 0.017 |
| 9 | 0.006 | 0.008 | 0.001 | 0.015 |
| 10 | 0.006 | 0.008 | 0.001 | 0.015 |
| 11 | 0.005 | 0.011 | 0.001 | 0.017 |
| 12 | 0.005 | 0.011 | 0.001 | 0.017 |
| 13 | 0.005 | 0.011 | 0.001 | 0.018 |
| 14 | 0.005 | 0.011 | 0.001 | 0.018 |
| 15 | 0.005 | 0.012 | 0.001 | 0.017 |
| 16 | 0.005 | 0.012 | 0.001 | 0.017 |
| 17 | 0.026 | 0.041 | 4.293 | 4.359 |
| 18 | 0.026 | 0.041 | 4.293 | 4.359 |
| 19 | 0.026 | 0.040 | 4.293 | 4.359 |
| 20 | 0.026 | 0.040 | 4.293 | 4.359 |
| 21 | 0.026 | 0.042 | 4.290 | 4.358 |
| 22 | 0.026 | 0.042 | 4.290 | 4.358 |
| 23 | 0.026 | 0.042 | 4.291 | 4.358 |
| 24 | 0.026 | 0.042 | 4.291 | 4.358 |
| 25 | 0.026 | 0.041 | 4.294 | 4.361 |
| 26 | 0.026 | 0.041 | 4.294 | 4.361 |
| 27 | 0.026 | 0.041 | 4.293 | 4.360 |
| 28 | 0.026 | 0.041 | 4.293 | 4.360 |
| 29 | 0.026 | 0.041 | 4.297 | 4.364 |
| 30 | 0.026 | 0.041 | 4.297 | 4.364 |
| 31 | 0.017 | 0.031 | 3.152 | 3.199 |
| 32 | 0.017 | 0.031 | 3.152 | 3.199 |
| 33 | 0.010 | 0.173 | 0.000 | 0.183 |
| 34 | 0.010 | 0.173 | 0.000 | 0.183 |
| 35 | 0.010 | 0.164 | 0.000 | 0.174 |
| 36 | 0.010 | 0.164 | 0.000 | 0.174 |
| 37 | 0.010 | 0.165 | 0.000 | 0.175 |
| 38 | 0.010 | 0.165 | 0.000 | 0.175 |
| 39 | 0.010 | 0.169 | 0.000 | 0.180 |
| 40 | 0.010 | 0.169 | 0.000 | 0.180 |
| 41 | 0.010 | 0.173 | 0.000 | 0.183 |
| 42 | 0.010 | 0.173 | 0.000 | 0.183 |
| 43 | 0.010 | 0.163 | 0.000 | 0.173 |
| 44 | 0.010 | 0.163 | 0.000 | 0.173 |
| 45 | 0.010 | 0.163 | 0.000 | 0.173 |
| 46 | 0.010 | 0.163 | 0.000 | 0.173 |
| 47 | 0.010 | 0.164 | 0.000 | 0.174 |
| 48 | 0.010 | 0.164 | 0.000 | 0.174 |
| 49 | 0.010 | 0.175 | 0.000 | 0.185 |
| 50 | 0.010 | 0.175 | 0.000 | 0.185 |
| 51 | 0.010 | 0.166 | 0.000 | 0.177 |
| 52 | 0.010 | 0.166 | 0.000 | 0.177 |
| 53 | 0.010 | 0.167 | 0.000 | 0.177 |
| 54 | 0.010 | 0.167 | 0.000 | 0.177 |
| 55 | 0.010 | 0.170 | 0.000 | 0.180 |
| 56 | 0.010 | 0.170 | 0.000 | 0.180 |
| 57 | 0.010 | 0.168 | 0.000 | 0.178 |
| 58 | 0.010 | 0.168 | 0.000 | 0.178 |
| 59 | 0.010 | 0.166 | 0.000 | 0.176 |
| 60 | 0.010 | 0.166 | 0.000 | 0.176 |
| 61 | 0.011 | 0.207 | 0.000 | 0.217 |
| 62 | 0.011 | 0.207 | 0.000 | 0.217 |
| 63 | 0.010 | 0.168 | 0.000 | 0.179 |
| 64 | 0.010 | 0.168 | 0.000 | 0.179 |
| 65 | 0.011 | 0.211 | 0.000 | 0.221 |
| 66 | 0.011 | 0.211 | 0.000 | 0.221 |
| 67 | 0.010 | 0.165 | 0.000 | 0.175 |
| 68 | 0.010 | 0.165 | 0.000 | 0.175 |
| 69 | 0.010 | 0.164 | 0.000 | 0.174 |
| 70 | 0.010 | 0.164 | 0.000 | 0.174 |
| 71 | 0.010 | 0.170 | 0.000 | 0.180 |
| 72 | 0.010 | 0.170 | 0.000 | 0.180 |
| 73 | 0.010 | 0.165 | 0.000 | 0.175 |
| 74 | 0.010 | 0.165 | 0.000 | 0.175 |
| 75 | 0.010 | 0.174 | 0.000 | 0.185 |
| 76 | 0.010 | 0.174 | 0.000 | 0.185 |
| 77 | 0.010 | 0.165 | 0.000 | 0.175 |
| 78 | 0.010 | 0.165 | 0.000 | 0.175 |
| 79 | 0.010 | 0.166 | 0.000 | 0.176 |
| 80 | 0.010 | 0.166 | 0.000 | 0.176 |
| tot | 0.957 | 9.003 | 66.424 | 76.384 |

Conf. 7

magnetization (x)

| # of ion | s | p | d | tot |
|---|---|---|---|---|
| 1 | 0.001 | -0.004 | 0.001 | -0.002 |
| 2 | 0.000 | -0.004 | 0.001 | -0.002 |
| 3 | 0.000 | -0.004 | 0.001 | -0.002 |
| 4 | 0.001 | -0.001 | 0.001 | 0.001 |
| 5 | 0.000 | -0.004 | 0.001 | -0.002 |
| 6 | 0.001 | -0.001 | 0.001 | 0.001 |
| 7 | 0.001 | -0.001 | 0.001 | 0.001 |
| 8 | 0.001 | -0.007 | 0.001 | -0.006 |
| 9 | -0.000 | 0.007 | -0.001 | 0.006 |
| 10 | -0.002 | 0.004 | -0.001 | 0.002 |
| 11 | -0.002 | 0.004 | -0.001 | 0.002 |
| 12 | 0.000 | 0.001 | -0.001 | -0.000 |
| 13 | -0.002 | 0.004 | -0.001 | 0.002 |
| 14 | 0.000 | 0.001 | -0.001 | -0.000 |
| 15 | 0.000 | 0.001 | -0.001 | -0.000 |
| 16 | -0.001 | 0.004 | -0.001 | 0.002 |
| 17 | 0.012 | 0.013 | 4.218 | 4.243 |
| 18 | 0.012 | 0.013 | 4.216 | 4.241 |
| 19 | 0.012 | 0.013 | 4.216 | 4.241 |
| 20 | 0.012 | 0.014 | 4.213 | 4.239 |
| 21 | 0.012 | 0.013 | 4.216 | 4.241 |
| 22 | 0.012 | 0.014 | 4.213 | 4.239 |
| 23 | 0.012 | 0.014 | 4.213 | 4.239 |
| 24 | -0.012 | -0.013 | -4.216 | -4.241 |
| 25 | -0.012 | -0.013 | -4.216 | -4.241 |
| 26 | -0.012 | -0.013 | -4.214 | -4.239 |
| 27 | -0.012 | -0.013 | -4.216 | -4.241 |
| 28 | -0.012 | -0.013 | -4.214 | -4.239 |
| 29 | -0.012 | -0.013 | -4.214 | -4.239 |
| 30 | -0.012 | -0.013 | -4.218 | -4.242 |
| 31 | -0.003 | -0.002 | -3.080 | -3.086 |
| 32 | 0.003 | 0.003 | 3.079 | 3.084 |
| 33 | 0.001 | 0.022 | 0.000 | 0.023 |
| 34 | 0.001 | 0.032 | 0.000 | 0.032 |
| 35 | 0.001 | 0.035 | 0.000 | 0.035 |
| 36 | 0.001 | 0.035 | 0.000 | 0.036 |
| 37 | 0.001 | 0.035 | 0.000 | 0.036 |
| 38 | 0.001 | 0.034 | 0.000 | 0.035 |
| 39 | 0.001 | 0.067 | 0.000 | 0.068 |
| 40 | 0.001 | 0.036 | 0.000 | 0.036 |
| 41 | 0.001 | 0.022 | 0.000 | 0.023 |
| 42 | 0.001 | 0.035 | 0.000 | 0.035 |
| 43 | 0.001 | 0.035 | 0.000 | 0.036 |
| 44 | 0.001 | 0.067 | 0.000 | 0.068 |
| 45 | 0.001 | 0.032 | 0.000 | 0.032 |
| 46 | 0.001 | 0.035 | 0.000 | 0.036 |
| 47 | 0.001 | 0.034 | 0.000 | 0.035 |
| 48 | 0.001 | 0.036 | 0.000 | 0.036 |
| 49 | 0.001 | 0.022 | 0.000 | 0.023 |
| 50 | 0.001 | 0.035 | 0.000 | 0.036 |
| 51 | 0.001 | 0.032 | 0.000 | 0.032 |
| 52 | 0.001 | 0.034 | 0.000 | 0.035 |
| 53 | 0.001 | 0.035 | 0.000 | 0.035 |
| 54 | 0.001 | 0.067 | 0.000 | 0.068 |
| 55 | 0.001 | 0.035 | 0.000 | 0.036 |
| 56 | 0.001 | 0.036 | 0.000 | 0.036 |
| 57 | -0.001 | -0.034 | 0.000 | -0.035 |
| 58 | -0.001 | -0.035 | 0.000 | -0.036 |
| 59 | -0.001 | -0.036 | 0.000 | -0.037 |
| 60 | -0.001 | -0.031 | 0.000 | -0.032 |
| 61 | -0.001 | -0.065 | 0.000 | -0.066 |
| 62 | -0.001 | -0.021 | 0.000 | -0.022 |
| 63 | -0.001 | -0.033 | 0.000 | -0.034 |
| 64 | -0.001 | -0.035 | 0.000 | -0.036 |
| 65 | -0.001 | -0.034 | 0.000 | -0.035 |
| 66 | -0.001 | -0.065 | 0.000 | -0.066 |
| 67 | -0.001 | -0.035 | 0.000 | -0.036 |
| 68 | -0.001 | -0.021 | 0.000 | -0.022 |
| 69 | -0.001 | -0.036 | 0.000 | -0.037 |
| 70 | -0.001 | -0.033 | 0.000 | -0.034 |
| 71 | -0.001 | -0.031 | 0.000 | -0.032 |
| 72 | -0.001 | -0.035 | 0.000 | -0.036 |
| 73 | -0.001 | -0.034 | 0.000 | -0.035 |
| 74 | -0.001 | -0.036 | 0.000 | -0.037 |
| 75 | -0.001 | -0.065 | 0.000 | -0.066 |
| 76 | -0.001 | -0.033 | 0.000 | -0.034 |
| 77 | -0.001 | -0.035 | 0.000 | -0.036 |
| 78 | -0.001 | -0.031 | 0.000 | -0.032 |
| 79 | -0.001 | -0.021 | 0.000 | -0.022 |
| 80 | -0.001 | -0.035 | 0.000 | -0.036 |

Conf. 8

magnetization (x)

| # of ion | s | p | d | tot |
|---|---|---|---|---|
| 1 | 0.000 | -0.003 | 0.001 | -0.002 |
| 2 | 0.001 | -0.004 | 0.001 | -0.001 |
| 3 | 0.001 | -0.004 | 0.001 | -0.001 |
| 4 | 0.015 | -0.006 | 0.001 | 0.010 |
| 5 | 0.001 | -0.004 | 0.001 | -0.001 |
| 6 | 0.015 | -0.006 | 0.001 | 0.010 |
| 7 | 0.015 | -0.006 | 0.001 | 0.010 |
| 8 | 0.002 | -0.007 | 0.001 | -0.004 |
| 9 | 0.004 | -0.002 | -0.001 | 0.001 |
| 10 | 0.003 | 0.004 | -0.000 | 0.007 |
| 11 | 0.003 | 0.004 | -0.000 | 0.007 |
| 12 | 0.002 | -0.001 | -0.001 | -0.001 |
| 13 | 0.003 | 0.004 | -0.000 | 0.007 |
| 14 | 0.002 | -0.001 | -0.001 | -0.001 |
| 15 | 0.002 | -0.001 | -0.001 | -0.001 |
| 16 | 0.000 | 0.005 | -0.001 | 0.005 |
| 17 | 0.012 | 0.014 | 4.221 | 4.246 |
| 18 | 0.014 | 0.017 | 4.230 | 4.261 |
| 19 | 0.014 | 0.017 | 4.230 | 4.261 |
| 20 | 0.013 | 0.019 | 4.227 | 4.259 |
| 21 | 0.014 | 0.017 | 4.230 | 4.261 |
| 22 | 0.013 | 0.019 | 4.227 | 4.259 |
| 23 | 0.013 | 0.019 | 4.227 | 4.259 |
| 24 | -0.012 | -0.014 | -4.213 | -4.240 |
| 25 | -0.012 | -0.014 | -4.213 | -4.240 |
| 26 | -0.012 | -0.014 | -4.215 | -4.241 |
| 27 | -0.012 | -0.014 | -4.213 | -4.240 |
| 28 | -0.012 | -0.014 | -4.215 | -4.241 |
| 29 | -0.012 | -0.014 | -4.215 | -4.241 |
| 30 | -0.011 | -0.013 | -4.210 | -4.235 |
| 31 | 0.015 | 0.027 | 3.145 | 3.187 |
| 32 | 0.005 | 0.003 | 2.788 | 2.797 |
| 33 | 0.010 | 0.176 | 0.000 | 0.185 |
| 34 | 0.001 | 0.039 | 0.000 | 0.040 |
| 35 | 0.001 | 0.041 | 0.000 | 0.042 |
| 36 | 0.001 | 0.038 | 0.000 | 0.039 |
| 37 | 0.001 | 0.044 | 0.000 | 0.045 |
| 38 | 0.001 | 0.063 | 0.000 | 0.064 |
| 39 | 0.000 | 0.019 | 0.000 | 0.019 |
| 40 | 0.001 | 0.047 | 0.000 | 0.048 |
| 41 | 0.010 | 0.176 | 0.000 | 0.185 |
| 42 | 0.001 | 0.041 | 0.000 | 0.042 |
| 43 | 0.001 | 0.044 | 0.000 | 0.045 |
| 44 | 0.000 | 0.019 | 0.000 | 0.019 |
| 45 | 0.001 | 0.039 | 0.000 | 0.040 |
| 46 | 0.001 | 0.038 | 0.000 | 0.039 |
| 47 | 0.001 | 0.063 | 0.000 | 0.064 |
| 48 | 0.001 | 0.047 | 0.000 | 0.048 |
| 49 | 0.010 | 0.176 | 0.000 | 0.185 |
| 50 | 0.001 | 0.044 | 0.000 | 0.045 |
| 51 | 0.001 | 0.039 | 0.000 | 0.040 |
| 52 | 0.001 | 0.063 | 0.000 | 0.064 |
| 53 | 0.001 | 0.041 | 0.000 | 0.042 |
| 54 | 0.000 | 0.019 | 0.000 | 0.019 |
| 55 | 0.001 | 0.038 | 0.000 | 0.039 |
| 56 | 0.001 | 0.047 | 0.000 | 0.048 |
| 57 | -0.001 | -0.020 | 0.000 | -0.020 |
| 58 | -0.000 | -0.015 | 0.000 | -0.016 |
| 59 | -0.000 | -0.026 | 0.000 | -0.026 |
| 60 | -0.001 | -0.024 | 0.000 | -0.024 |
| 61 | 0.010 | 0.192 | 0.000 | 0.202 |
| 62 | -0.002 | -0.062 | 0.000 | -0.064 |
| 63 | -0.000 | -0.021 | 0.000 | -0.021 |
| 64 | -0.000 | -0.014 | 0.000 | -0.014 |
| 65 | -0.001 | -0.020 | 0.000 | -0.020 |
| 66 | 0.010 | 0.192 | 0.000 | 0.202 |
| 67 | -0.000 | -0.015 | 0.000 | -0.016 |
| 68 | -0.002 | -0.062 | 0.000 | -0.064 |
| 69 | -0.000 | -0.026 | 0.000 | -0.026 |
| 70 | -0.000 | -0.021 | 0.000 | -0.021 |
| 71 | -0.001 | -0.024 | 0.000 | -0.024 |
| 72 | -0.000 | -0.014 | 0.000 | -0.014 |
| 73 | -0.001 | -0.020 | 0.000 | -0.020 |
| 74 | -0.000 | -0.026 | 0.000 | -0.026 |
| 75 | 0.010 | 0.192 | 0.000 | 0.202 |
| 76 | -0.000 | -0.021 | 0.000 | -0.021 |
| 77 | -0.000 | -0.015 | 0.000 | -0.016 |
| 78 | -0.001 | -0.024 | 0.000 | -0.024 |
| 79 | -0.002 | -0.062 | 0.000 | -0.064 |
| 80 | -0.000 | -0.014 | 0.000 | -0.014 |

Conf. 9

magnetization (x)

| # of ion | s | p | d | tot |
|---|---|---|---|---|
| 1 | 0.004 | 0.012 | 0.001 | 0.017 |
| 2 | 0.004 | 0.012 | 0.001 | 0.017 |
| 3 | 0.004 | 0.012 | 0.001 | 0.017 |
| 4 | 0.005 | 0.008 | 0.001 | 0.015 |
| 5 | 0.004 | 0.012 | 0.001 | 0.017 |
| 6 | 0.005 | 0.008 | 0.001 | 0.015 |
| 7 | 0.005 | 0.008 | 0.001 | 0.015 |
| 8 | 0.004 | 0.010 | 0.001 | 0.015 |
| 9 | 0.004 | 0.009 | 0.001 | 0.014 |
| 10 | 0.005 | 0.011 | 0.001 | 0.018 |
| 11 | 0.005 | 0.011 | 0.001 | 0.018 |
| 12 | 0.004 | 0.009 | 0.001 | 0.014 |
| 13 | 0.005 | 0.011 | 0.001 | 0.018 |
| 14 | 0.004 | 0.009 | 0.001 | 0.014 |
| 15 | 0.004 | 0.009 | 0.001 | 0.014 |
| 16 | 0.004 | 0.012 | 0.001 | 0.018 |
| 17 | 0.026 | 0.041 | 4.295 | 4.362 |
| 18 | 0.026 | 0.041 | 4.293 | 4.360 |
| 19 | 0.026 | 0.041 | 4.293 | 4.360 |
| 20 | 0.026 | 0.041 | 4.291 | 4.359 |
| 21 | 0.026 | 0.041 | 4.293 | 4.360 |
| 22 | 0.026 | 0.041 | 4.291 | 4.359 |
| 23 | 0.026 | 0.041 | 4.291 | 4.359 |
| 24 | 0.026 | 0.041 | 4.292 | 4.359 |
| 25 | 0.026 | 0.041 | 4.292 | 4.359 |
| 26 | 0.026 | 0.041 | 4.293 | 4.360 |
| 27 | 0.026 | 0.041 | 4.292 | 4.359 |
| 28 | 0.026 | 0.041 | 4.293 | 4.360 |
| 29 | 0.026 | 0.041 | 4.293 | 4.360 |
| 30 | 0.026 | 0.041 | 4.295 | 4.362 |
| 31 | 0.016 | 0.031 | 3.149 | 3.196 |
| 32 | 0.016 | 0.031 | 3.147 | 3.195 |
| 33 | 0.010 | 0.171 | 0.000 | 0.181 |
| 34 | 0.010 | 0.166 | 0.000 | 0.176 |
| 35 | 0.010 | 0.165 | 0.000 | 0.176 |
| 36 | 0.010 | 0.168 | 0.000 | 0.178 |
| 37 | 0.010 | 0.165 | 0.000 | 0.175 |
| 38 | 0.010 | 0.166 | 0.000 | 0.176 |
| 39 | 0.010 | 0.199 | 0.000 | 0.210 |
| 40 | 0.010 | 0.168 | 0.000 | 0.178 |
| 41 | 0.010 | 0.171 | 0.000 | 0.181 |
| 42 | 0.010 | 0.165 | 0.000 | 0.176 |
| 43 | 0.010 | 0.165 | 0.000 | 0.175 |
| 44 | 0.010 | 0.199 | 0.000 | 0.210 |
| 45 | 0.010 | 0.166 | 0.000 | 0.176 |
| 46 | 0.010 | 0.168 | 0.000 | 0.178 |
| 47 | 0.010 | 0.166 | 0.000 | 0.176 |
| 48 | 0.010 | 0.168 | 0.000 | 0.178 |
| 49 | 0.010 | 0.171 | 0.000 | 0.181 |
| 50 | 0.010 | 0.165 | 0.000 | 0.175 |
| 51 | 0.010 | 0.166 | 0.000 | 0.176 |
| 52 | 0.010 | 0.166 | 0.000 | 0.176 |
| 53 | 0.010 | 0.165 | 0.000 | 0.176 |
| 54 | 0.010 | 0.199 | 0.000 | 0.210 |
| 55 | 0.010 | 0.168 | 0.000 | 0.178 |
| 56 | 0.010 | 0.168 | 0.000 | 0.178 |
| 57 | 0.010 | 0.164 | 0.000 | 0.174 |
| 58 | 0.010 | 0.166 | 0.000 | 0.177 |
| 59 | 0.010 | 0.168 | 0.000 | 0.178 |
| 60 | 0.010 | 0.165 | 0.000 | 0.175 |
| 61 | 0.011 | 0.199 | 0.000 | 0.210 |
| 62 | 0.010 | 0.171 | 0.000 | 0.181 |
| 63 | 0.010 | 0.165 | 0.000 | 0.175 |
| 64 | 0.010 | 0.167 | 0.000 | 0.178 |
| 65 | 0.010 | 0.164 | 0.000 | 0.174 |
| 66 | 0.011 | 0.199 | 0.000 | 0.210 |
| 67 | 0.010 | 0.166 | 0.000 | 0.177 |
| 68 | 0.010 | 0.171 | 0.000 | 0.181 |
| 69 | 0.010 | 0.168 | 0.000 | 0.178 |
| 70 | 0.010 | 0.165 | 0.000 | 0.175 |
| 71 | 0.010 | 0.165 | 0.000 | 0.175 |
| 72 | 0.010 | 0.167 | 0.000 | 0.178 |
| 73 | 0.010 | 0.164 | 0.000 | 0.174 |
| 74 | 0.010 | 0.168 | 0.000 | 0.178 |
| 75 | 0.011 | 0.199 | 0.000 | 0.210 |
| 76 | 0.010 | 0.165 | 0.000 | 0.175 |
| 77 | 0.010 | 0.166 | 0.000 | 0.177 |
| 78 | 0.010 | 0.165 | 0.000 | 0.175 |
| 79 | 0.010 | 0.171 | 0.000 | 0.181 |
| 80 | 0.010 | 0.167 | 0.000 | 0.178 |

| | | | | | | | | | | | | | | |
|---|---|---|---|---|---|---|---|---|---|---|---|---|---|---|
| tot | 0.001 | 0.012 | -0.001 | 0.012 | tot | 0.160 | 1.461 | 6.026 | 7.648 | tot | 0.955 | 8.996 | 66.412 | 76.363 |
